# Using protein-per-mRNA differences among human tissues in codon optimization

**DOI:** 10.1186/s13059-023-02868-2

**Published:** 2023-02-24

**Authors:** Xavier Hernandez-Alias, Hannah Benisty, Leandro G. Radusky, Luis Serrano, Martin H. Schaefer

**Affiliations:** 1 Centre for Genomic Regulation (CRG), The Barcelona Institute of Science and Technology, Dr. Aiguader 88, 08003 Barcelona, Spain; 2 Universitat Pompeu Fabra (UPF), 08002 Barcelona, Spain; 3 ICREA, Pg. Lluís Companys 23, 08010 Barcelona, Spain; 4 IEO European Institute of Oncology IRCCS, Department of Experimental Oncology, Via Adamello 16, 20139 Milan, Italy

**Keywords:** Tissue, Codon optimization, Gene design, Translation, Proteomics, Transcriptomics

## Abstract

**Background:**

Codon usage and nucleotide composition of coding sequences have profound effects on protein expression. However, while it is recognized that different tissues have distinct tRNA profiles and codon usages in their transcriptomes, the effect of tissue-specific codon optimality on protein synthesis remains elusive.

**Results:**

We leverage existing state-of-the-art transcriptomics and proteomics datasets from the GTEx project and the Human Protein Atlas to compute the protein-to-mRNA ratios of 36 human tissues. Using this as a proxy of translational efficiency, we build a machine learning model that identifies codons enriched or depleted in specific tissues. We detect two clusters of tissues with an opposite pattern of codon preferences. We then use these identified patterns for the development of CUSTOM, a codon optimizer algorithm which suggests a synonymous codon design in order to optimize protein production in a tissue-specific manner. In human cell-line models, we provide evidence that codon optimization should take into account particularities of the translational machinery of the tissues in which the target proteins are expressed and that our approach can design genes with tissue-optimized expression profiles.

**Conclusions:**

We provide proof-of-concept evidence that codon preferences exist in tissue-specific protein synthesis and demonstrate its application to synthetic gene design. We show that CUSTOM can be of benefit in biological and biotechnological applications, such as in the design of tissue-targeted therapies and vaccines.

**Supplementary Information:**

The online version contains supplementary material available at 10.1186/s13059-023-02868-2.

## Background

From the advent of synthetic biology, it is widely recognized that gene design needs to be adapted to the expression requirements of the host [1]. Within coding sequences, there are manifold overlapping factors that determine translation, mRNA stability, transcription, splicing, methylation, or ribosomal frameshifting, among others [2]. Therefore, while the amino acid sequence of proteins is maintained, the usage of synonymous codons can be optimized for heterologous expression.

During the last decades, an extensive number of computational tools have been developed for gene design [3, 4]. Most commonly, these tools optimize the codon usage in order to resemble that of the host based on the Codon Adaptation Index (CAI) of the genes to be optimized or similar metrics. Other more innovative developments also include neural networks that control translation speed [5] or other machine learning algorithms that optimize mRNA stability [6]. Although there is no absolute “best” approach, codon optimization is commonly and successfully applied in gene design. In fact, current knowledge on the effect of synonymous variants on the heterologous expression of the protein GFP shows up to 46-fold expression differences in HeLa cells [7]. Similarly, mRNA and protein levels across thousands of GFP variants strongly correlated with their CAI in *S. cerevisiae* [8].

Nevertheless, codon optimization in multicellular eukaryotes is more intricately determined, since different tissues can showcase differences in codon usage and tRNA expression [9–11]. The translational efficiency, which constitutes the rate of protein production from mRNA, is therefore dependent on the balance between the codon usage of genes being translated and the abundance of a limited tRNA pool [10, 12]. In this context, codons translated by highly abundant tRNAs generally correspond to optimal codons in the translatome, as has been reported by ribosome profiling [13]. However, detecting differences of translational efficiency between tissues can be challenging, since the larger gene-to-gene variability of protein levels can obscure the actual tissue-to-tissue differences [14].

The advent of high-throughput sequencing has enabled an extensive transcriptome profiling of human tissues [15, 16]. Based on the mRNA-seq data from the GTEx project, Kames et al. (2020) developed the public resource TissueCoCoPUTs, containing codon and codon pair usage tables of tissue transcriptomes [11]. However, current knowledge indicates that tissue-specific variability of gene expression is mostly regulated at the post-transcriptional level and mRNA-seq alone is therefore not able to capture it [17, 18]. Developments in mass spectrometry have very recently led to the release of deep and quantitative proteome maps of human tissues [19, 20].

Using this transcriptomic and proteomic data from the Human Protein Atlas and the GTEx project, we here compute the protein-to-mRNA (PTR) ratios of 36 human tissues as a proxy for translational efficiency. To distinguish high-PTR from low-PTR proteins, we build random forest models that identify which codons are optimal or non-optimal for each tissue. Then, we apply these codon preferences to develop a tool, CUSTOM (Codon Usage to Specific Tissue OptiMizer), that optimizes coding sequences for a specific tissue. CUSTOM is publicly available as a Python package (https://github.com/hexavier/CUSTOM) and as a web interface (https://custom.crg.eu). By optimizing eGFP and mCherry proteins to a human cell model of kidney and lung, we provide experimental evidence of how tissue codon optimization could be important, e.g., in vaccines or gene therapy.

## Results

### Protein-to-mRNA ratios detect differences in translational efficiency among tissues

**Fig. 1:**
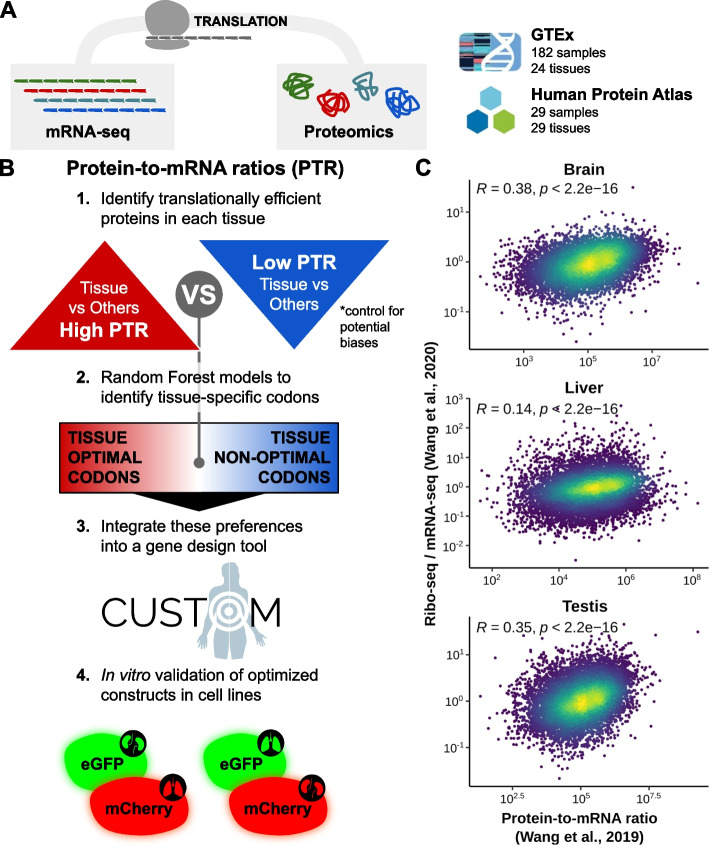
Protein-to-mRNA ratios detect differences in translational efficiency among tissues. **A** Proteomics and mRNA-seq data included in this study contains samples from the GTEx project [19] and Human Protein Atlas [20]. **B** Using these datasets, we compute the protein-to-mRNA ratios (PTR) and define tissue-enriched and tissue-depleted sets of proteins for each tissue. By comparing the codon usage of these two sets, we identify the codon optimality pattern of tissues. Using this information, we develop a gene design tool called CUSTOM and validate the method using an in vitro cellular model. **C** Spearman correlation between the median translational efficiency [21] (ratio between ribo-seq and mRNA-seq FPKMs) and PTR [20] across genes in the brain, liver, and testis. The color code depicts the density of points in the scatter plot

Translational efficiency (TE) is defined as the rate of protein synthesis from mRNAs, which can be approximately estimated as the protein-to-mRNA (PTR) ratio. To systematically analyze the PTR ratios across a total of 36 human tissues, we retrieved the mRNA-seq and proteomics data from two recent datasets: 29 tissues from the Human Protein Atlas [17, 20] (HPA) and 24 tissues from the GTEx project [19] (Fig. 1A, B, Additional file 2). The first study includes one sample per tissue, which are concurrently analyzed by mRNA-seq and label-free iBAQ proteomics. On the latter, a total of 182 matched samples are measured both by mRNA-seq and tandem mass tag 10plex/MS3 mass spectrometry. By correlating the mRNA expression, protein abundance and PTR ratios along the 17 tissues in common, we could ascertain a significant correspondence between the two datasets (Additional file 1: Fig. S1A).

Although to date this data is still relatively rare, a more direct readout of TE is the ratio between ribosome profiling and mRNA abundance. To confirm the validity of using PTR ratios as an estimate of TE, we therefore compared the PTR values to a ribosome profiling dataset of the brain, liver, and testis. In all of them, we observed a significantly positive correlation across the human genome [21] (Fig. 1C, Additional file 2).

We next set out to investigate the tissue-to-tissue differences of PTR ratios in the aforementioned datasets. For each tissue, we defined a set of high-PTR and a set of low-PTR genes, described as having a PTR fold change compared to the average of all other tissues larger than 2, and vice versa (see Methods, Additional file 2). We found a significant concordance between the gene sets derived from the HPA and GTEx datasets in most tissues (*p* < 0.05, one-tailed binomial test, Additional file 2). In particular, 16/17 tissues were significantly concordant at the mRNA level, 15/17 at the proteomic level, and 9/17 at the PTR level; only colon tissue was disagreeing at all levels.

To physiologically interpret the differences between gene sets, we performed an enrichment map among high-PTR and low-PTR sets linking tissues with high overlap of the respective gene sets (Additional file 1: Fig. S1B). In agreement with their highly tissue-specific function, we detected that tissues group according to their role in the body: e.g., nervous tissue (brain and tibial nerve), muscular tissue (skeletal muscle and heart). Moreover, GO analyses of high-PTR genes showed significant enrichments for highly tissue-specific biological processes according to the physiological and anatomical function of the tissue (*p* < 0.05, Fisher’s exact test, Additional file 1: Fig. S1C).

We next asked if there could be any confounding factors associated with these gene sets, such as protein secretion and protein or mRNA degradation, that could bias our analyses. First, it was recently reported that constitutively secreted proteins are often detected at the mRNA but not at the protein level [19], which could bias PTR ratios as a measure of TE. While we also observed these differences in our dataset (Additional file 1: Fig. S2A), the exclusion of secreted proteins from our gene sets did not affect the downstream results (see following section). Second, we analyzed the protein half-life of gene sets based on two recent datasets in five human cell lines [22, 23] (Additional file 2). The protein half-life was not significantly different between high-PTR and low-PTR gene sets in most of the tissues (*p* < 0.05, two-tailed Wilcoxon rank-sum test), nor was there any trend that one of the groups would be consistently associated with higher or lower half-life (Additional file 1: Fig. S2B). Similarly, no consistent association with mRNA degradation was detected using the mRNA half-life measurements of three different cell lines [24–26] either (Additional file 1: Fig. S2C, Additional file 2).

Taken together, these observations indicate that PTR ratios can efficiently detect tissue-specific differences in translation. As such, it constitutes an appropriate dataset to systematically study TE differences across the set of 36 human tissues.

### Random forest models identify two clusters of human tissues with distinct codon signatures

Recent studies show that different tissues can have different tRNA repertoires and codon usage [10, 11], which could have an influence on translational efficiency. Therefore, we wondered whether high-PTR and low-PTR sets of genes were specifically enriched or depleted of certain codons. If there is a tissue-specific codon signature, we would expect to be able to predict these differences in PTR.

**Fig. 2:**
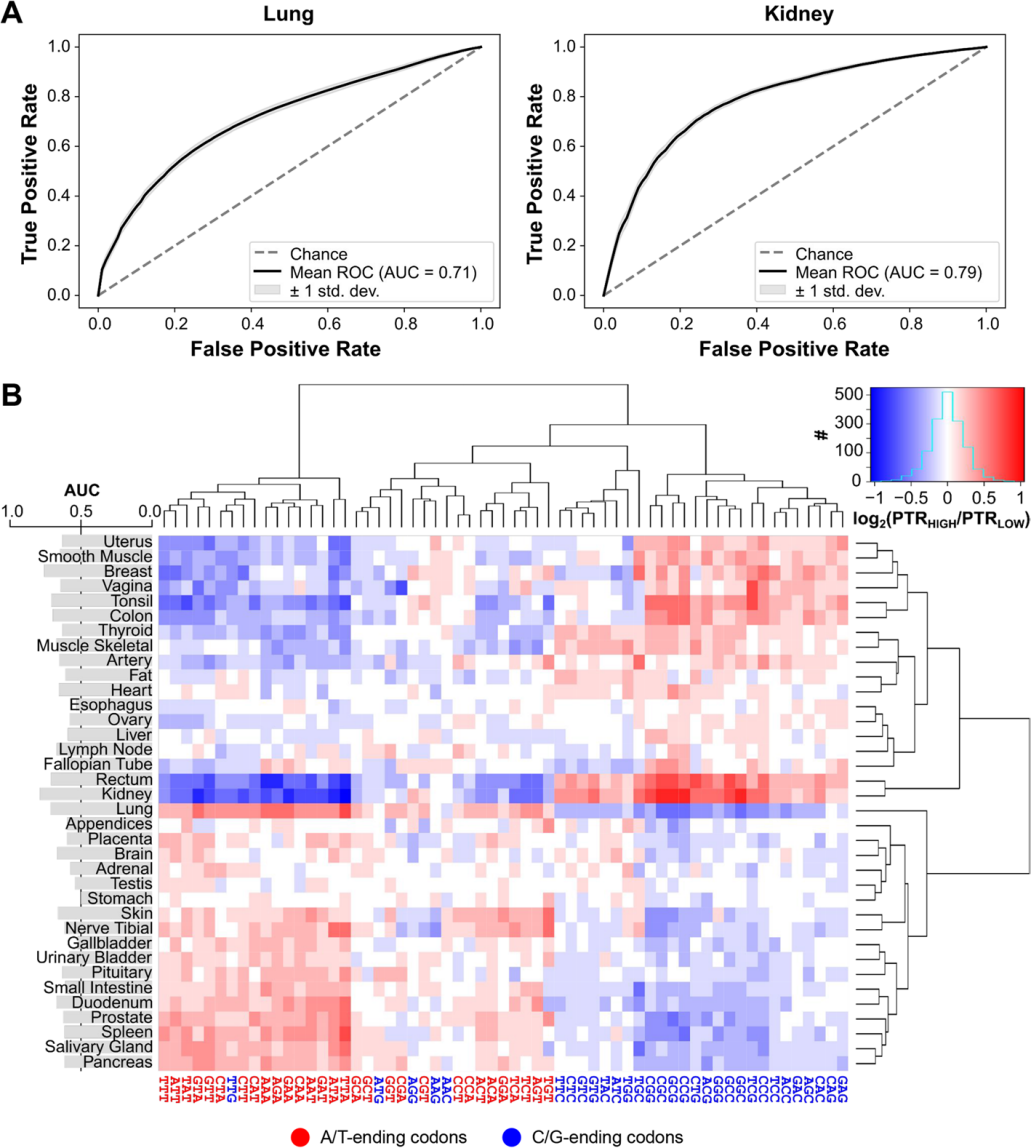
Random Forest models identify two clusters of human tissues with distinct codon signatures. **A** Receiver operating characteristic (ROC) curves of lung and kidney random forest classifiers, in which the codon usage of genes is used to predict whether they are high-PTR or low-PTR in the respective tissue (see the “Methods” section). **B** Ratios of the codon usage between high-PTR and low-PTR genes in each tissue. Codons and tissues are hierarchically clustered using Euclidean distances and the complete-linkage method. The barplot on the left shows the mean AUC of the ROC curve of the RF model of each tissue

To that aim, we built a random forest classifier for each tissue that predicts the high-PTR vs low-PTR state of genes based on their codon usage. All 36 resulting models performed with an area under the curve (AUC) of their receiver operating characteristic (ROC) curves higher than the no-skill model of 0.5 (Fig. 2A, Additional file 3). In particular, kidney, breast, lung, rectum, and tonsil showcased the highest tissue-specific profiles (Fig. 2A; all AUC > 0.70). Furthermore, to validate whether these differences in PTR were specifically dependent on codon usage and not from nucleotide composition alone, we compared them with the performance of three control models: +1 and +2 misframed codon usage as well as dinucleotide composition of genes (Additional file 3). While these control models also showed predictive power, the AUC of the correctly framed codon usage models significantly outperformed the controls (*p* < 0.05, one-tailed binomial test).

To examine the tissue-specificity of codons, we next analyzed which particular codons were predictive for high vs low PTR states in each tissue. The relative feature importances of each random forest classifier measure the contribution of codons in the decision trees (Additional file 1: Fig. S3A). In general, only a few codons (5 to 10) were relevant for each model, but they differed across tissues. A recursive feature elimination of each model similarly substantiated that fewer than 10 codons were sufficient to achieve the maximum AUC performance (Additional file 1: Fig. S3B).

In addition, by computing the ratio between the codon usage of high-PTR vs low-PTR genes, we observed the enrichment or depletion of codons in specific tissues (Fig. 2B). There were two main clusters of tissues with opposite codon optimality profiles: the first generally preferring A/T-ending codons while the second favoring C/G-ending ones. In agreement with studies showing that the A/T-vs-G/C pattern is associated with proliferation [10, 27], we also detected that the A/T-ending cluster was significantly more proliferative than G/C-ending one, based on the proliferation marker Ki67 [28] (Additional file 1: Fig. S3C). Also, as expected, tissues with higher AUC performances showcased more definite codon profile patterns both in terms of their enrichment/depletion (Fig. 2B) as well as their importance (Additional file 1: Fig. S3A). As mentioned in the previous section, we also repeated the same analyses with the secretome-excluded sets of genes, which had a highly similar codon optimality profile with all correlations of codon ratios over 0.95 (Additional file 3).

To further assess the concordance between GTEx and HPA datasets, we also reproduced the same analyses with the high-PTR vs low-PTR sets of each dataset separately (Additional file 2). With few differences, both datasets showed a similar A/T-ending vs G/C-ending signature across tissues (Additional file 1: Fig. S4, Additional file 3). Moreover, to disentangle the codon effects on mRNA and protein levels separately, we constructed a similar model using tissue-specific sets from either mRNA-seq or proteomic measurements alone (Additional file 2). As expected, since PTRs are defined as protein divided by transcript abundance, we observed an inverted codon signature related to mRNAs (Additional file 1: Fig. S5A, Additional file 3). Conversely, proteomics data alone led to a similar but less pronounced codon pattern as compared to PTRs (Additional file 1: Fig. S5B, Additional file 3), indicating that both mRNAs and proteins contribute to the observed PTR differences.

Given that some reports highlight the role of codon pair bias in translation [11, 29], we similarly analyzed the codon pair usage ratios between high-PTR vs low-PTR genes (Additional file 4). A principal component analysis (PCA) of these ratios perfectly separated the exact same two clusters observed above with single codons alone (Additional file 1: Fig. S6A). To further analyze how much codon pair variance was explained by single codons alone, we compared observed codon pair ratios with their expected values based on their constituent single codons. They related highly linearly as shown by linear regression models (Additional file 1: Fig. S6B, Additional file 4), which indicates that differences in codon pair ratios can be explained by single codons alone. In fact, codon pairs that deviated the most from linearity just corresponded to outliers with very low counts within gene sets (Additional file 1: Fig. S6C).

Overall, our random forest classifiers can predict the PTR of genes in a certain tissue based on their codon usage. As such, the observed differences in codon preference or avoidance across tissues can be exploited to optimize tissue-specific gene design.

### CUSTOM generates fluorescent variants with desired tissue-specific expression

To translate differences in tissue-specific PTR into a codon optimizer tool, we developed CUSTOM as a probabilistic approach (see Methods, https://custom.crg.eu). Given a certain amino acid sequence and a target tissue, codons are selected with a probability proportional to their tissue importance in the model (Additional file 1: Fig. S3A). Then, based on the ratio of the selected codon (Fig. 2B), it is either added or avoided in the generated sequence. This process is performed along the whole sequence and repeated iteratively to generate a pool of hundreds of optimized sequences. Among this pool of sequences, given that tissue-specific codon usage is not the only factor influencing coding sequences [2], the top scoring ones can be selected based on other commonly used parameters of codon bias or mRNA stability [4] (Codon Adaptation Index, Codon Pair Bias, Minimum Free Energy, Effective Number of Codons, see the “Methods” section).

**Fig. 3:**
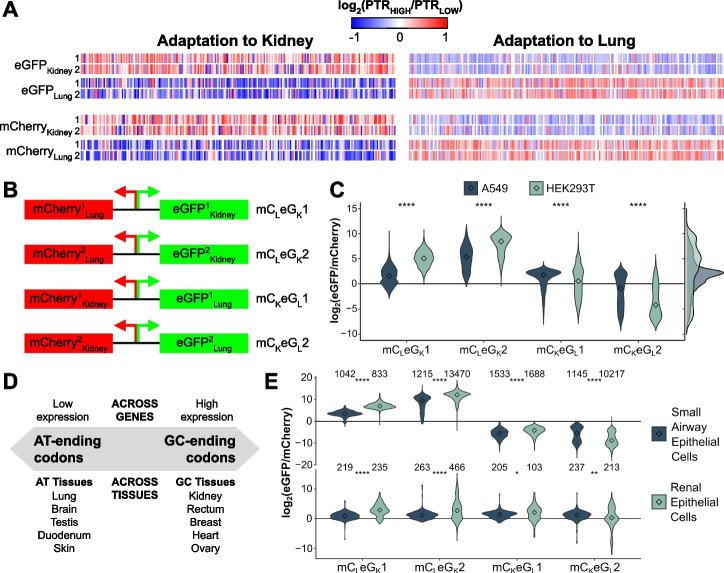
CUSTOM generates fluorescent variants with desired tissue-specific expression. **A** Selected eGFP and mCherry sequences optimized to lung and kidney using CUSTOM. The color code corresponds to the optimality ratios of Fig. 2B. **B** Using these sequences, we designed four of constructs by placing a mCherry and an eGFP with opposite tissue-specificity under an inducible bidirectional promoter. **C** Ratios of eGFP and mCherry for each of the four constructs in A549 and HEK293T cell lines, detected by flow cytometry. Three biological replicates are downsampled to 1000 cells per group and summed; see individual replicates in Additional file 1: Fig. S7E. On the right, the distribution of all four constructs together are shown. **D** Conceptual summary of the relationship between the gene codon usage and their expression across tissues. **E** Ratios of eGFP and mCherry for each of the four constructs in primary cells, detected by flow cytometry. Top and bottom panels correspond to two independent batches of primary cells (see the “Methods” section). The number of cells within each group is specified. Center values represent the median. Statistical differences were determined by two-tailed Wilcoxon rank-sum test and are denoted as follows: *p ≤ 0.05, **p ≤ 0.01, ***p ≤ 0.001, ****p ≤ 0.0001

To validate the predictor, we chose the proteins eGFP and mCherry, and optimized them with CUSTOM to either kidney or lung (Additional file 5). Taking eight among the top optimized sequences (Fig. 3A, 2x eGFP_Kidney_, 2x eGFP_Lung_, 2x mCherry_Kidney_, 2x mCherry_Lung_), we then designed four constructs, placing in each of them one eGFP and one mCherry optimized each one for a different tissue and under an inducible bidirectional promoter (Fig. 3B, mC_L_eG_K_1, mC_L_eG_K_2, mC_K_eG_L_1, mC_K_eG_L_2). These constructs were then simultaneously expressed in the lung and kidney cell lines A549 and HEK293T, respectively. Based on available proteomics data of these cell lines [30], both the proteome and codon usage of A549 clearly resembled that of lung, while HEK293T was a closer model to kidney (Additional file 1: Fig. S7A-D).

We then analyzed the eGFP and mCherry fluorescence of each construct in each cell line by flow cytometry (Additional file 6). For all cases, we observed that the eGFP/mCherry ratio was significantly higher in the tissue for which eGFP was optimized (Fig. 3C, *p* < 0.05, two-tailed Wilcoxon rank-sum test), which validated our tissue-specificity hypothesis. Moreover, while the efficiency of transfection was extremely variable between replicates, cell lines, and constructs, we detected consistent significant differences in all three replicates (Additional file 1: Fig. S7E). Quantification of eGFP and mCherry by targeted proteomics showed the same tissue-specific pattern (Additional file 1: Fig. S8A-B, Additional file 5). We further observed that (1) the two constructs with eGFP_Lung_ had generally lower eGFP/mCherry ratios compared to the ones with eGFP_Kidney_, and (2) the differences in eGFP/mCherry ratios between constructs were more variable in HEK293T than A549 cells (Fig. 3C, right panel). Altogether, these observations suggest that A/T-ending codons are generally lower expressed than C/G-ending counterparts, but tissues like lung tolerate them better (Fig. 3D, Additional file 1: Fig. S8B). Furthermore, to confirm that observed changes arise from differences in mRNA translation rather than at the transcript level, we quantified eGFP and mCherry transcript abundances by RT-qPCR (Additional file 5). In agreement, measured mRNA ratios inversely corresponded with protein ratios for three of the constructs (Additional file 1: Fig. S8C), which concurs with the previously observed mRNA model (Additional file 1: Fig. S5A).

As A549 is a cancer cell line and HEK293T is of adrenal origin [31], we sought to validate the construct expression in a more physiological model of tissue primary cells. The same tissue-specific pattern was consistently observed for all constructs except mC_K_eG_L_1 (where differences were less evident), both by flow cytometry (Fig. 3E, Additional file 7) and targeted proteomics (Additional file 1: Fig. S8D-E, Additional file 5). Altogether, the results reported in this section validate our PTR-derived models and provide proof-of-principle experimental evidence for tissue-specific codon optimization using CUSTOM.

## Discussion

Current analyses of the mRNA and protein levels among human tissues distinguish between across-gene and within-gene (i.e., across-tissue) variability [14]. In fact, the coefficient of variation of mRNA and protein levels across genes highly exceeds that of across tissues. In consequence, studies of codon usage on human transcriptomes and PTR ratios so far were dominated by the across-gene variability and thus overlooked the smaller across-tissue differences [11, 17]. The approach taken here puts the focus on the across-tissue variability of PTR ratios rather than the overall genome, which is actually the major source of post-transcriptional regulation [17, 18]. In fact, we provide evidence that high-PTR gene sets of tissues are particularly enriched for tissue-specific functions.

Given the high GC content of the human genome as a whole, G/C-ending codons are generally more abundant (i.e., higher CAI) and relate to higher mRNA and protein expression levels [7, 32, 33]. But again, moving away from this across-gene perspective of human codon usage to look at the across-tissue variation, we here report that distinct tissues showcase different codon preferences. All in all, as also determined experimentally, we observe that the expression of a certain protein is dependent on two axes: (1) the across-gene axis with G/C-ending codons favoring higher absolute expression and (2) the tissue-specific axis with the codon preferences observed in Fig. 2B. Moreover, we also report that some tissues have a more definite codon profile than others, where this second axis is less evident. In agreement with our observed tissue-specific axis, Allen et al. (2022) recently reported that testis and brain (in contrast to other tissues such as ovary) better tolerate the translation of rare A/T-ending codons in *Drosophila melanogaster* [34].

The codon optimization tool CUSTOM is able to exploit these codon preferences for the design of tissue-targeted genes. In fact, the designed constructs expressed in kidney and lung cell lines and primary cells showed the predicted tissue-specificity. To make CUSTOM readily available to the community, we developed it completely open source and made it accessible through a web server.

Human tissues are ensembles of heterogeneous cell types and therefore observed differences in codon optimality are actually a composition of the constituent cell types. However, single-cell technologies of mRNA and protein measurements fall still far from complete cellular atlases [35]. Instead, we used the most up-to-date and complete tissue-wide maps of the human transcriptome and proteome, which have been generated by cutting-edge mass spectrometry and mRNA sequencing techniques [15, 19, 20]. Moreover, while PTRs positively correlated with TE and results were not confounded by factors such as secretion or mRNA and protein degradation, more direct readouts of TE will be possible as more ribosome profiling datasets of human tissues become available.

## Conclusions

The results presented here constitute a proof-of-concept that tissue-specific codon usage exists and can be applied to gene design. In particular, this tool could be used in the development of optimized gene therapies or mRNA vaccines with more defined tissue targets and therefore potentially less side effects. Nevertheless, factors other than codon usage also play a role in gene expression [2], and therefore changes in synonymous codons can as well interfere with other processes such as mRNA folding and stability, mRNA modifications, protein folding, or translational kinetics [36, 37]. As such, tissue-specific codon usage will constitute one additional instrument in the gene design tool set.

## Methods

### Codon optimizer for tissue-specific expression

CUSTOM is implemented in Python (version > = 3.7) and available on GitHub (https://github.com/hexavier/CUSTOM) and as a web interface (https://custom.crg.eu). The landscape of possible synonymous sequences is vast and manifold factors overlap in defining the code. Therefore, we follow a simple probabilistic approach with two steps: (1) translate tissue-specific codon preferences into a pool of optimal sequences and (2) select the desired sequence based on other parameters of relevance.

#### Creating a pool of tissue-optimized sequences

The algorithm requires two main input data: the amino acid sequence to be optimized (or DNA sequence) and the target tissue. For each iteration of the optimization, the sequence is optimized taking two factors into account: how important the codon is in defining tissue-specificity (relative feature weights in Additional file 1: Fig. S3A) and whether it is enriched or depleted in the tissue (codon ratios in Fig. 2B). Therefore, for each amino acid, a certain codon is selected with a probability proportional to the first. If the selected codon is enriched in the tissue, it is incorporated into the sequence. If it is depleted, the codon is excluded and another codon is selected based on the same probabilities as before. This process is repeated along the full sequence and for as many iterations as desired. Furthermore, given that 5–10 top codons are often sufficient to achieve the full AUC prediction (Additional file 1: Fig. S3B), users can also control whether optimizing all codons or only the top ones.

#### Selecting the top scoring candidates

Once a pool of optimized sequences has been generated, the best-ranked ones can be selected as the user desires. Given that no ground truth is known, the default *select_best* method of the package measures a list of standard metrics frequently used in gene design and computes an average to select the top scoring sequences. The following factors can be included:

- Minimum Free Energy (MFE): a measure of mRNA stability from the ViennaRNA package [38]. CUSTOM distinguishes between the first 40 nucleotides (whose weak secondary structure leads to increased translation initiation) and the rest of the sequence (whose strong secondary structure relates to longer mRNA half-lives) [4]
- Codon Adaptation Index (CAI): a measure of similarity between the codon usage of the sequence and that of the human genome [39]
- Codon Pair Bias (CPB): a measure of similarity between the codon pair usage of the sequence and that of the human genome [29]
- Effective Number of Codons (ENC): a measure of codon evenness. A value of 20 means that all 100% codons are biased towards the most common codon, while 61 corresponds to no bias at all [40]
- GC content: a measure of similarity between the sequence GC content and a desired target value of GC
- Homopolymers: filters out sequences with homopolymers of a certain length, which can lead to worse expression
- Motifs: filters out sequences containing certain motifs

### Experimental model and protocol

#### Human cell models

The cell lines included in this study are HEK293T and A549. The sex of each cell line is as follows: HEK293T, female; A549, male. Cells were maintained at 37 °C in a humidified atmosphere at 5% CO_2_ in DMEM 4.5 g/l Glucose with UltraGlutamine media supplemented with 10% of FBS and 1% penicillin/streptomycin. Primary epithelial cells were obtained from Lonza. Both renal (Lonza, cat # CC-2556) and small airway (Lonza, cat # CC-2547) cells were grown at 37 °C in the corresponding growth media (Lonza, cat # CC-3190 and CC-3118). Two independent batches of primary cells were used for each cell type. Small airway cells were obtained from two female donors (batch #1: 18TL082942, 68 years; batch #2: 18TL179344, 25 years), and renal cells were obtained from a male donor (batch #1 and #2: 19TL036410).

#### Expression vectors design

We applied CUSTOM to the protein sequences of eGFP and mCherry (Uniprot ID: C5MKY7, X5DSL3). Sequences were optimized to either lung or kidney, generating a total of *n_pool* = 1000. Sequences with homopolymers equal or larger than 7 were filtered out and scored with:

$$opt. select\_ best\left(by=\left\{^\prime\kern-.1em{^\prime}\kern-.2em{MFE}^\prime\kern-.1em{^\prime}:{}^\prime\kern-.1em{^\prime}\kern-.2em{min}^\prime\kern-.1em{^\prime},{}^\prime\kern-.1em{^\prime}\kern-.2em{MFEini}^\prime\kern-.1em{^\prime}:{}^\prime\kern-.1em{^\prime}\kern-.2em{max}^\prime\kern-.1em{^\prime},{}^\prime\kern-.1em{^\prime}\kern-.2em{CAI}^\prime\kern-.1em{^\prime}:{}^\prime\kern-.1em{^\prime}\kern-.2em{max}^\prime\kern-.1em{^\prime},{}^\prime\kern-.1em{^\prime}\kern-.2em{CPB}^\prime\kern-.1em{^\prime}:{}^\prime\kern-.1em{^\prime}\kern-.2em{max}^\prime\kern-.1em{^\prime},{}^\prime\kern-.1em{^\prime}\kern-.2em{ENC}^\prime\kern-.1em{^\prime}:{}^\prime\kern-.1em{^\prime}\kern-.2em{min}^\prime\kern-.1em{^\prime}\right\}, homopolymers=7, top=10\right)$$

Among the top 10 scoring candidates of each optimization, we selected 2x eGFP_Kidney_, 2x eGFP_Lung_, 2x mCherry_Kidney_, and 2x mCherry_Lung_ (Additional file 5).

For gene overexpression experiments, the two selected eGFP and and mCherry were cloned into a modified version of the XLone-GFP vector (Addgene, cat # 96930). The modification consisted of replacing the promoter of XLone-GFP with a bidirectional TRE3G promoter (Clontech), which allows the simultaneous expression of both genes. The four constructs consisted in a combination of eGFP_Lung_ + mCherry_Kidney_ and eGFP_Kidney_ + mCherry_Lung_.

#### Flow cytometry

HEK293T and A549 cells were seeded in 6-well plates in technical triplicates for each condition and biological replicate. Expression vectors were transfected with Lipofectamine 3000 (Invitrogen). Similarly, primary renal and small airway cells were seeded in 6-well plates in technical triplicates for each condition and biological replicate and expression vectors were transfected using TransfeX (ATCC). Gene expression was induced with 500 ng/mL of doxycycline during 48 h. To measure the expression of the fluorescent proteins, cells were trypsinized and resuspended with 500 μL of media. Samples were applied on a FACS Fortessa analyzer. Approximately 10^4^ live single-cell events were collected per sample. BD FACSDiva software was used for gating and analysis. The fluorescence intensity for each population in the FITC channel and PE–Texas Red channel was obtained.

#### Targeted proteomics

HEK293T and A549 cells were seeded in 6-well plates. Expression vectors were transfected with Lipofectamine 3000 (Invitrogen). Similarly, primary renal and small airway cells were seeded in 6-well plates and expression vectors were transfected using TransfeX (ATCC). Gene expression was induced with 500 ng/mL of doxycycline during 48h. To measure the expression of the fluorescent proteins, cells were washed twice with PBS and resuspended in 6M Urea/200mM ABC buffer.

##### Sample preparation

Samples (10 μg) were reduced with dithiothreitol (30 nmol, 37 °C, 60 min) and alkylated in the dark with iodoacetamide (60 nmol, 25 °C, 30 min). The resulting protein extract was first diluted to 2M urea with 200 mM ammonium bicarbonate for digestion with endoproteinase LysC (1:10 w:w, 37°C, 6h, Wako, cat # 129-02541) and then diluted 2-fold with 200 mM ammonium bicarbonate for trypsin digestion (1:10 w:w, 37°C, o/n, Promega, cat # V5113).

After digestion, peptide mix was acidified with formic acid and desalted with a MicroSpin C18 column (The Nest Group, Inc) prior to LC-MS/MS analysis.

##### Chromatographic and mass spectrometric analysis

Samples were analyzed using an Orbitrap Lumos (Thermo Fisher Scientific) coupled to an EASY-nanoLC 1200 UPLC system (Thermo Fisher Scientific). Peptides were loaded directly onto the analytical column and were separated by reversed-phase chromatography using a 50-cm column with an inner diameter of 75 μm, packed with 2 μm C18 particles spectrometer (Thermo Scientific, San Jose, CA, USA).

Chromatographic gradients started at 95% buffer A and 5% buffer B with a flow rate of 300 nl/min for 5 minutes and gradually increased to 25% buffer B and 75% A in 79 min and then to 40% buffer B and 60% A in 11 min. After each analysis, the column was washed for 10 min with 10% buffer A and 90% buffer B. Buffer A: 0.1% formic acid in water. Buffer B: 0.1% formic acid in 80% acetonitrile.

The mass spectrometer was operated in positive ionization mode with an EASY-Spray nanosource at 2.4kV and at a source temperature of 305 °C.

##### Library data

The acquisition was performed in data-dependent acquisition (DDA) mode and full MS scans with 1 micro scans at resolution of 120,000 were used over a mass range of m/z 350-1400 with detection in the Orbitrap mass analyzer. Auto gain control (AGC) was set to “standard” and injection time to “auto.” In each cycle of data-dependent acquisition analysis, following each survey scan, the most intense ions above a threshold ion count of 10000 were selected for fragmentation. The number of selected precursor ions for fragmentation was determined by the “Top Speed” acquisition algorithm and a dynamic exclusion of 60 s. Fragment ion spectra were produced via high-energy collision dissociation (HCD) at normalized collision energy of 28%, and they were acquired in the ion trap mass analyzer. AGC was set to 2E4, and an isolation window of 0.7 m/z and a maximum injection time of 12 ms were used.

Digested bovine serum albumin (New England Biolabs, cat # P8108S) was analyzed between each sample to avoid sample carryover and to assure stability of the instrument and QCloud [41] has been used to control instrument longitudinal performance during the project.

Acquired spectra were analyzed using the Proteome Discoverer software suite (v2.5, Thermo Fisher Scientific) and the Mascot search engine (v2.6, Matrix Science [42]). The data were searched against a Swiss-Prot human database (as of March 2021, 20386 entries) plus eGFP, mCherry, a list [43] of common contaminants and all the corresponding decoy entries. For peptide identification, a precursor ion mass tolerance of 7 ppm was used for MS1 level, trypsin was chosen as enzyme, and up to three missed cleavages were allowed. The fragment ion mass tolerance was set to 0.5 Da for MS2 spectra. Oxidation of methionine and N-terminal protein acetylation were used as variable modifications whereas carbamidomethylation on cysteines was set as a fixed modification. False discovery rate (FDR) in peptide identification was set to a maximum of 1%.

The best three peptides of mCheery and eGFP were used in the PRM method.

##### PRM data

A full MS scan with 1 micro scans at resolution of 30,000 was used over a mass range of m/z 350–1400 with detection in the Orbitrap mass analyzer. A PRM (parallel reaction monitoring) method was used for data acquisition with a quadrupole isolation window set to 1.4 m/z and MSMS scans over a mass range of m/z 300–2000, with detection in the Orbitrap at resolution of 120,000. MSMS fragmentation was performed using HCD at 30 NCE; the auto gain control (AGC) was set to 1e5 and maximum injection time of 118 ms. Peptide masses (m/z) were defined in the mass list table for further fragmentation (Table 1).

**Table 1:** Peptide masses (m/z) defined in the PRM method for further fragmentation

Peptide and gene name	m/z	***z***
FSVSGEGEGDATYGK_GFP	752.3335	2
EDGNILGHK_GFP	491.7513	2
FEGDTLVNR_GFP	525.7644	2
KPVQLPGAYNVNIK_mCherry	514.2997	3
LDITSHNEDYTIVEQYER_mCherry	742.3501	3
HSTGGMDELYK_mCherry	619.2795	2

The Skyline software [44] (v20.2.1.278) was used to generate the libraries (observed as the output of the DDA data Proteome Discoverer (v2.5) search and predicted with Prosit [45]) and extract the fragment areas of each peptide.

### RT-qPCR

HEK293 and A549 cells were seeded in 6-well plates in technical replicates for each condition. Expression vectors were transfected with Lipofectamine 3000 (Invitrogen) and gene expression was induced with 500 ng/mL of doxycycline during 48h. RNA isolation was performed with the RNeasy micro kit (Qiagen). eGFP and mCherry transcript abundances were quantified by RT-qPCR (Power SYBR Green RNA-to-CT 1-Step Kit, ThermoFisher). Primers for mC_L_eG_K_1: GFPfwd 5′-GAGCTCAAGGGCATCGACTT-3′, GFPrev 5′-CTGCTGGTAATGGTCTGCCA-3′, mCherryfwd 5′-AGGTGCACATGGAAGGAAGT-3′, mCherryrev 5′-TGTGGGGAGAGTATGTCCCAT-3′. Primers for mC_L_eG_K_2: GFPfwd 5′-TACGTGCAGGAACGGACAAT-3′, GFPrev 5′-GATGTTGCCGTCCTCCTTGA-3′, mCherryfwd 5′-GGGAAGCATCTAGCGAACGA-3′, mCherryrev 5′-CACCAGGTAGTTGAACAGGCT-3′. Primers for mC_K_eG_L_1: GFPfwd 5′-TGCCGTGGCCTACTTTAGTT-3′, GFPrev 5′-TACGTACCCTTCGGGCATTG-3′, mCherryfwd 5′-GACGCCGAGGTGAAAACAAC-3′, mCherryrev 5′-TGTAGTCCTCGTTGTGCGAC-3′. Primers for mC_K_eG_L_2: GFPfwd 5′-GGATGGAGACGTGAACGGAC-3′, GFPrev 5′-AAGGCACTGGTAGCTTTCCT-3′, mCherryfwd 5′-AAGCTGAAGGTGACGAAGGG-3′, mCherryrev 5′-TCGAAGTTCATCACCCGCTC-3′. As both eGFP and mCherry genes were in the same expression cassette, for each sample, the Ct values of eGFP were normalized to the Ct values of mCherry, ΔCt =(Ct_eGFP_ -Ct_mCherry_) and represented as 2^−ΔCt^.

### Data sources

#### Protein-to-mRNA ratios

The PTR ratios of the HPA were directly retrieved from the Table EV3 of Eraslan et al. (2019) [17]. In this dataset, protein levels are determined as absolute abundances based on their iBAQ quantification. As for the GTEx data, we retrieved protein and mRNA levels from the supplement of Jiang et al. (2020) [19], respectively. In this case, the proteomics measurements are relative quantifications from a tandem mass tag (TMT) 10plex/MS3 mass spectrometry strategy. To compute their PTR ratios, we followed the same pipeline as in the HPA: (1) proteins with an abundance of 0 were considered as missing values (NA); (2) protein quantifications were adjusted to have in each tissue the same median than the overall median; (3) genes with a TPM lower than 10 were taken as non-transcribed (NA). With that, comparable PTR values between HPA and GTEx are obtained (Additional file 1: Fig. S1A).

#### Codon and codon pair usage tables

The codon usage and codon pair usage tables of *Homo sapiens* from RefSeq were downloaded from the Codon/Codon Pair Usage Tables (CoCoPUTs) project release as of June 9, 2020 [46]. Regarding the codon usage of misframed coding sequences and their dinucleotide composition, we computed them from the latest release of the CCDS database of human sequences (release 22) [47].

#### Translational efficiencies

The processed data of matched ribosome profiling and mRNA-seq samples from the brain, liver, and testis was retrieved from ArrayExpress (E-MTAB-7247) [21]. Translational efficiencies were then computed as the ratio FPKM_Ribo-seq_/FPKM_mRNA-seq_.

#### Protein half-life

The log-10-transformed protein half-lives for B cells, NK cells, hepatocytes, monocytes, and HeLa cells were downloaded from Eraslan et al. (2019) [17] (https://github.com/EraslanBas/HumanTransProt), which includes data from two studies [22, 23]. Given the concordance of half-lives among the five cell types (Additional file 2), we used their average for the analysis in this work (Additional file 1: Fig. S2B).

#### mRNA half-life

The log-10-transformed protein half-lives for HEK293, HeLa, and K562 cells were downloaded from Eraslan et al. (2019) [17] (https://github.com/EraslanBas/HumanTransProt), which includes data from three studies [24–26]. Given the concordance of half-lives among the three cell types (Additional file 2), we used their average for the analysis in this work (Additional file 1: Fig. S2C).

#### Blood secretome

Using the predictions by the HPA [16], there are 2641 secretome genes, 729 of which are secreted to blood. Given that we were concerned on proteins that are not detected at the protein levels because of their systemic rather than local secretion, we focused our analysis on the latter (Additional file 2).

### Computational analysis

#### High-PTR and low-PTR gene sets

As PTR values from GTEx were computed from relative TMT proteomics in contrast to the absolute iBAQ quantification of HPA, they were not directly comparable and thus we defined the high-PTR and low-PTR gene sets for each dataset separately. On the one hand, high-PTR genes fulfilled three conditions: (1) genes having a PTR fold change compared to the average of all other tissues larger than 2, (2) genes with the highest PTR among all tissues, (3) genes detected in at least 3 tissues in the dataset. On the other hand, low-PTR genes were defined as: (1) genes having a PTR fold change compared to the average of all other tissues smaller than 0.5, (2) genes with the lowest PTR among all tissues, (3) genes detected in at least 3 tissues in the dataset. As a result, we defined one high-PTR and one low-PTR gene set for each tissue in each dataset. For those 17 tissues in common between both HPA and GTEx datasets, the union between both datasets was taken except for genes with contradictory labels, which were excluded.

The same three conditions were used to define the high-mRNA/low-mRNA and the high-protein/low-protein gene sets (Additional file 1: Fig. S5). With these, we followed the same subsequent steps as with PTR sets.

#### Random forest classifiers

To identify the most important codons determining high-PTR vs low-PTR genes, we computed their codon usage normalized by length, so that all 61 amino-acid-encoding codons sum up to 1. Taking this table of normalized codon usage as features, we applied a random forest (RF) classifier, populated with 100 decision trees, using the scikit-learn package [48]. Therefore, for each of the 36 tissues, we developed a model for predicting the high-PTR vs low-PTR genes based on their codon usage. To control for size differences between high-PTR and low-PTR groups, we iteratively sampled equal-sized groups, for *n* = 100 iterations. Furthermore, we validated the results with a stratified 5-fold cross-validation. In order to evaluate the performance of the RF models, we computed the area under the curve (AUC) of receiver operating characteristic (ROC) plots (Fig. 2A). We took the average and standard deviation across all iterations. Similarly, we computed the relative feature weights corresponding to each of the 61 codons (Fig. 2B).

To validate that the predictive potential of RF classifiers were codon-specific, we similarly computed the length-normalized codon usage of + 1 and + 2 misframed coding sequences as well as dinucleotide usage. By running the exact same pipeline as above, we determined the average AUC of these three control RF classifiers (Additional file 3). We used a one-tailed binomial test to analyze whether the AUCs of controls were lower than the original model more often than expected by chance (*p* = 1/2).

While the relative feature weights determine the importance of each codon in distinguishing high-PTR vs low-PTR genes, they do not provide any directionality. To analyze whether codons are enriched or depleted in high-PTR vs low-PTR genes, we computed the ratios between the average length-normalized codon usage of high-PTR and low-PTR genes. Similarly, codon pair ratios were computed in the same way.

Among the total of 61 amino-acid-encoding codons, we also analyzed how many of them were actually informative in the models using a recursive feature elimination (RFE). Therefore, for each tissue, we started by building a full model with all 61 codons and then recursively removed the least important one, as determined by the relative feature weights, until only one was left. At each step, we computed the AUC of the ROC curve of the model as explained above (Additional file 1: Fig. S3B).

#### Enrichment map

For this analysis, in order to allow an overlap between tissue gene sets, we used a slightly less stringent tissue-specificity definition. High-PTR sets were defined as (1) genes having a PTR fold change compared to the average of all other tissues larger than 2 and (2) genes detected in at least 3 tissues in the dataset, and vice versa for low-PTR sets.

To analyze the overlap between tissue gene sets, we used the EnrichmentMap app from Cytoscape [49]. We defined a generic input of high-PTR and low-PTR sets of proteins per tissue. Similarity was computed as the overlap coefficient ([size of (A intersect B)]/[size of (minimum(A ,B))]).

#### Gene Ontology enrichment analysis

Gene Ontology (GO) categories of Biological Processes were analyzed for enrichment as of May 27, 2021 [50]. Enrichment analyses were performed by PANTHER using the Fisher’s exact test and Bonferroni correction for multiple testing [51].

#### Principal component analysis of codon pairs

We applied principal component analysis to the codon pair ratios of each tissue in order to explore the main variability among tissues along the 4096 codon pair ratios.

#### Linear regression of codon pairs

We fitted a linear regression model between the observed codon ratios (dependent variable) and the expected ratios based on single codons alone (independent variable). The expected values were computed as the product of the ratios of the two codons that constitute the pair. For each model, we computed the R squared, the residual standard error (RSE), and the model *p*-value (Additional file 4).

#### Statistical analysis

All details of the statistical analyses can be found in the “Results” section and the figure legends. We used a significance level of 0.05.

## Supplementary Information

**Additional file 1: Supplementary figures. Fig. S1.** Protein-to-mRNA ratios detect differences in translational efficiency among tissues. **Fig. S2.** Differences in secretion, protein half-life and mRNA half-life among tissues. **Fig. S3.** Random Forest models identify two clusters of human tissues with distinct codon signatures. **Fig. S4.** Random Forest models of HPA and GTEx datasets independently. **Fig. S5.** Random Forest models of tissue-specific mRNA and protein levels. **Fig. S6.** Differences between tissues are also observed at the codon pair level. **Fig. S7.** CUSTOM generates fluorescent variants with desired tissue-specific expression. **Fig. S8.** Proteomics and RT-qPCR of CUSTOM-optimized constructs.

**Additional file 2. **Protein-to-mRNA ratios among tissues. (A) Protein-to-mRNA ratios of the Human Protein Atlas. (B) Protein-to-mRNA ratios of GTEx. (C) GTEx samples. (D) Translational efficiency of the brain, liver and testis. (E) High-PTR and low-PTR sets of proteins for each tissue. (F) Concordance of protein sets between tissues in both HPA and GTEx. (G) Protein half-lives of five different human cell lines. (H) mRNA half-lives of three different human cell lines. (I) Proteins secreted to blood. https://doi.org/10.6084/m9.figshare.21379197.

**Additional file 3. **Random Forest models of codon usage. (A) Results of RF models for each of the 36 tissues. (B) Codon ratios between high-PTR vs low-PTR genes for each tissue. (C) Codon ratios between high-PTR vs low-PTR genes for each tissue, after excluding secretome proteins. (D) Random Forest models based on codon usage of misframed CDSs and dinucleotide usage. (E) Codon ratios between high-PTR vs low-PTR genes for each tissue of HPA. (F) Codon ratios between high-PTR vs low-PTR genes for each tissue of GTEx. (G) Codon ratios between high-mRNA vs low-mRNA genes for each tissue. (H) Codon ratios between high-protein vs low-protein genes for each tissue. https://doi.org/10.6084/m9.figshare.21379194.

**Additional file 4. **Models of codon pair usage. (A) Codon pair ratios between high-PTR vs low-PTR proteins for each tissue. (B) Linear regression between observed vs expected codon pair ratios. https://doi.org/10.6084/m9.figshare.21379200.

**Additional file 5. **CUSTOM-optimized fluorescent variants. (A) Relative Codon Usage of optimized protein variants. (B) Sequences of optimized protein variants. (C) Targeted proteomics of GFP and mCherry peptides. (D) RT-qPCR of GFP and mCherry transcripts. https://doi.org/10.6084/m9.figshare.21407610.

**Additional file 6. **Flow cytometry of cell lines. The eGFP and mCherry fluorescence is detected by flow cytometry for each of the four constructs transfected into HEK293T and A549 cell lines. https://doi.org/10.6084/m9.figshare.21379206.

**Additional file 7. **Flow cytometry of primary cells. The eGFP and mCherry fluorescence is detected by flow cytometry for each of the four constructs transfected into Small Airway Epithelial Cells and Renal Epithelial Cells. https://doi.org/10.6084/m9.figshare.21379209.

**Additional file 8. **Peer review history.

## Data Availability

The code used in this study is available at GitHub (https://github.com/hexavier/codon_optimization, MIT LICENSE) [52], and the CUSTOM software is available as a Python package (https://github.com/hexavier/CUSTOM, GNU GENERAL PUBLIC LICENSE v3.0) [53] and a web interface (https://custom.crg.eu). All datasets and code generated or analyzed during this study are available at Figshare (10.6084/m9.figshare.c.6260508) [54]. The mass spectrometry proteomics data have been deposited to the ProteomeXchange Consortium via the PRIDE [55] partner repository with the dataset identifier PXD037866 [56].
