# Supplementary material for: Using protein-per-mRNA differences among human tissues in codon optimization
Source: Genome Biol. 2023 Feb 24;24:34. doi: 10.1186/s13059-023-02868-2 (PMC9951436; doi:10.1186/s13059-023-02868-2)
Supplement: Supplementary file 1 — Additional file 1: Supplementary figures. Fig. S1. Protein-to-mRNA ratios detect differences in translational efficiency among tissues. Fig. S2. Differences in secretion, protein half-life and mRNA half-life among tissues. Fig. S3. Random Forest models identify two clusters of human tissues with distinct codon signatures. Fig. S4. Random Forest models of HPA and GTEx datasets independently. Fig. S5. Random Forest models of tissue-specific mRNA and protein levels. Fig. S6. Differences between tissues are also observed at the codon pair level. Fig. S7. CUSTOM generates fluorescent variants with desired tissue-specific expression. Fig. S8. Proteomics and RT-qPCR of CUSTOM-optimized constructs. [file 13059_2023_2868_MOESM1_ESM.pdf]

SUPPLEMENTARY FIGURES

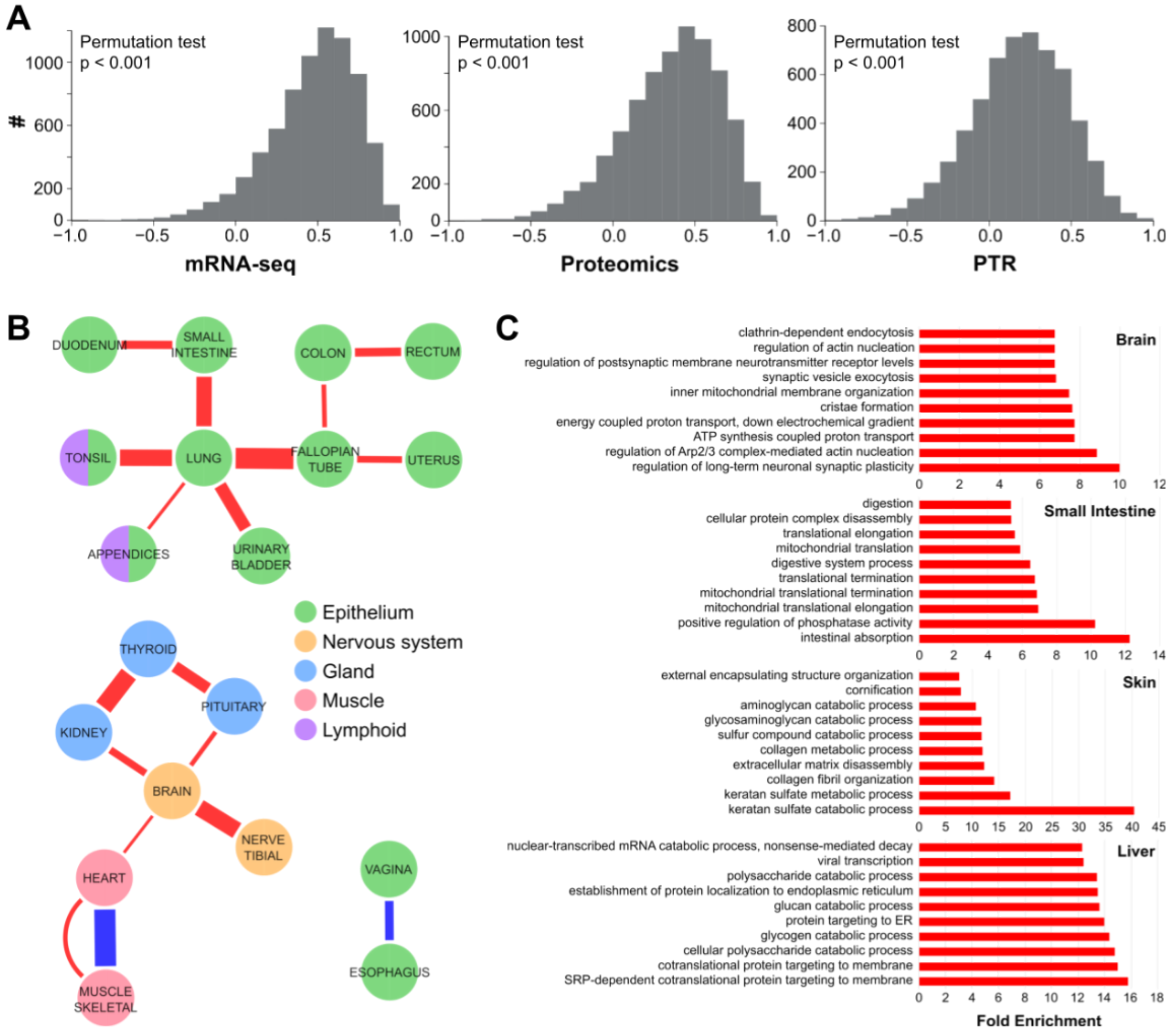

Figure S1. Protein-to-mRNA ratios detect differences in translational efficiency among tissues, related to Figure 1. (A) Correspondence between GTEx and Human Protein Atlas datasets at the mRNA-seq, proteomics and PTR levels. The histograms show the Spearman correlation of each gene along all 17 tissues in common between both datasets. Only correlations of genes detected in more than 5 tissues were computed. Significance is determined by assessing median(R_{real} - $R_{permuted}$) > 0 for a total of 1000 tissue permutations. (B) Enrichment Map of high-PTR (red) and low-PTR (blue) sets of proteins among tissues. Edges show significant enrichments between sets with a similarity coefficient >0.33. The width of edges is proportional to the similarity coefficient. The color of nodes depicts their tissue type based on the BRENDA Tissue Ontology [1]. Tissues with no significant edges are not shown. (C) GO enrichment analysis of biological processes for the high-PTR sets of four tissues. The top 10 significant GO terms with a Bonferroni-corrected p-value ≤ 0.05 are shown.

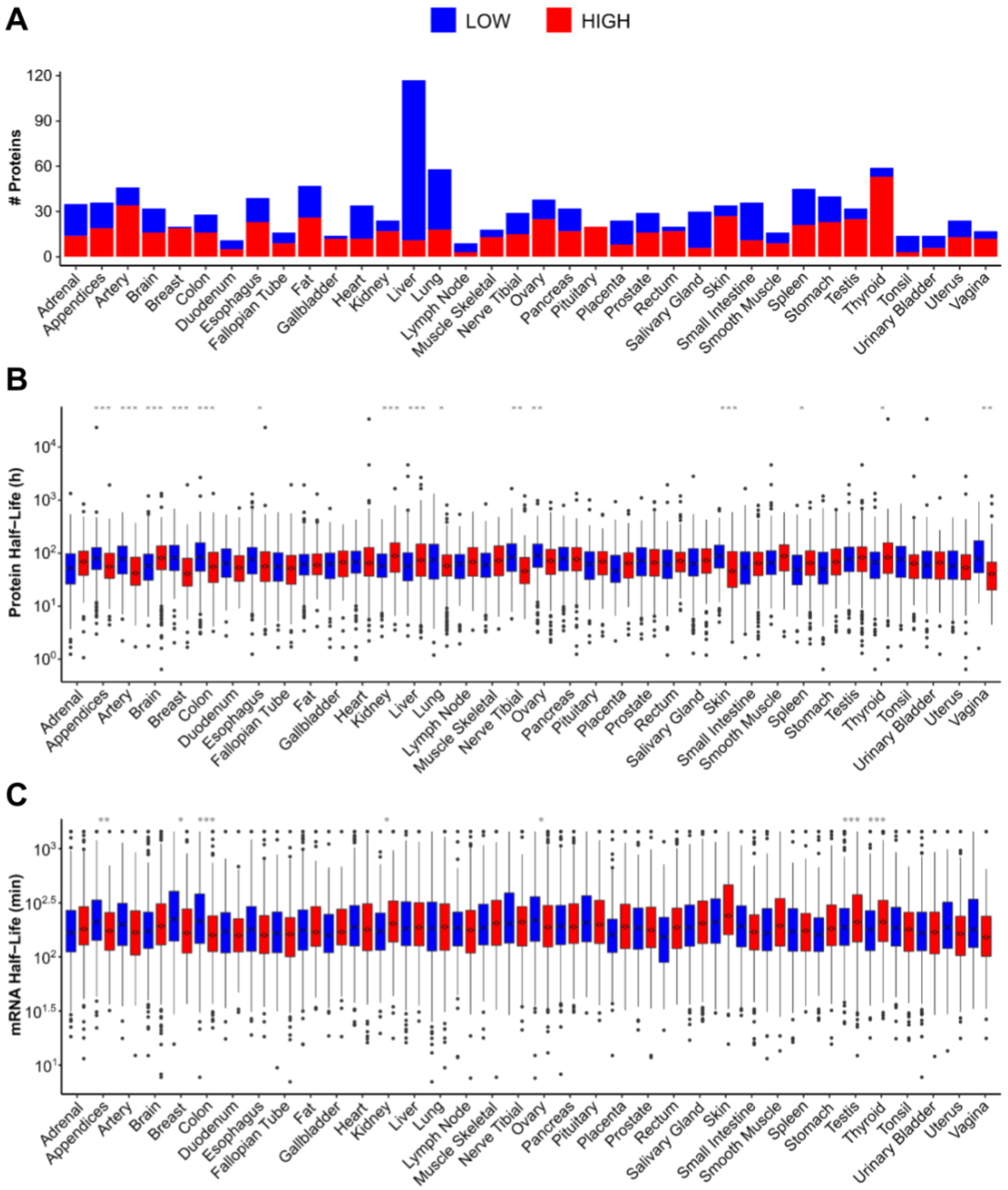

Figure S2. Differences in secretion, protein half-life and mRNA half-life among tissues, related to Figure 1. (A) Number of proteins secreted to blood in the low-PTR and high-PTR sets of genes in each tissue. (B) Average protein half-life of low-PTR and high-PTR sets of genes per tissue. (C) Average mRNA half-life of low-PTR and high-PTR sets of genes per tissue. Statistical differences were determined by two-tailed Wilcoxon rank-sum test and corrected for multiple comparisons using the Holm-Bonferroni method. Only significant differences are shown and are denoted as follows: $^*p \leq 0.05$, $^{**}p \leq 0.01$, $^{***}p \leq 0.001$.

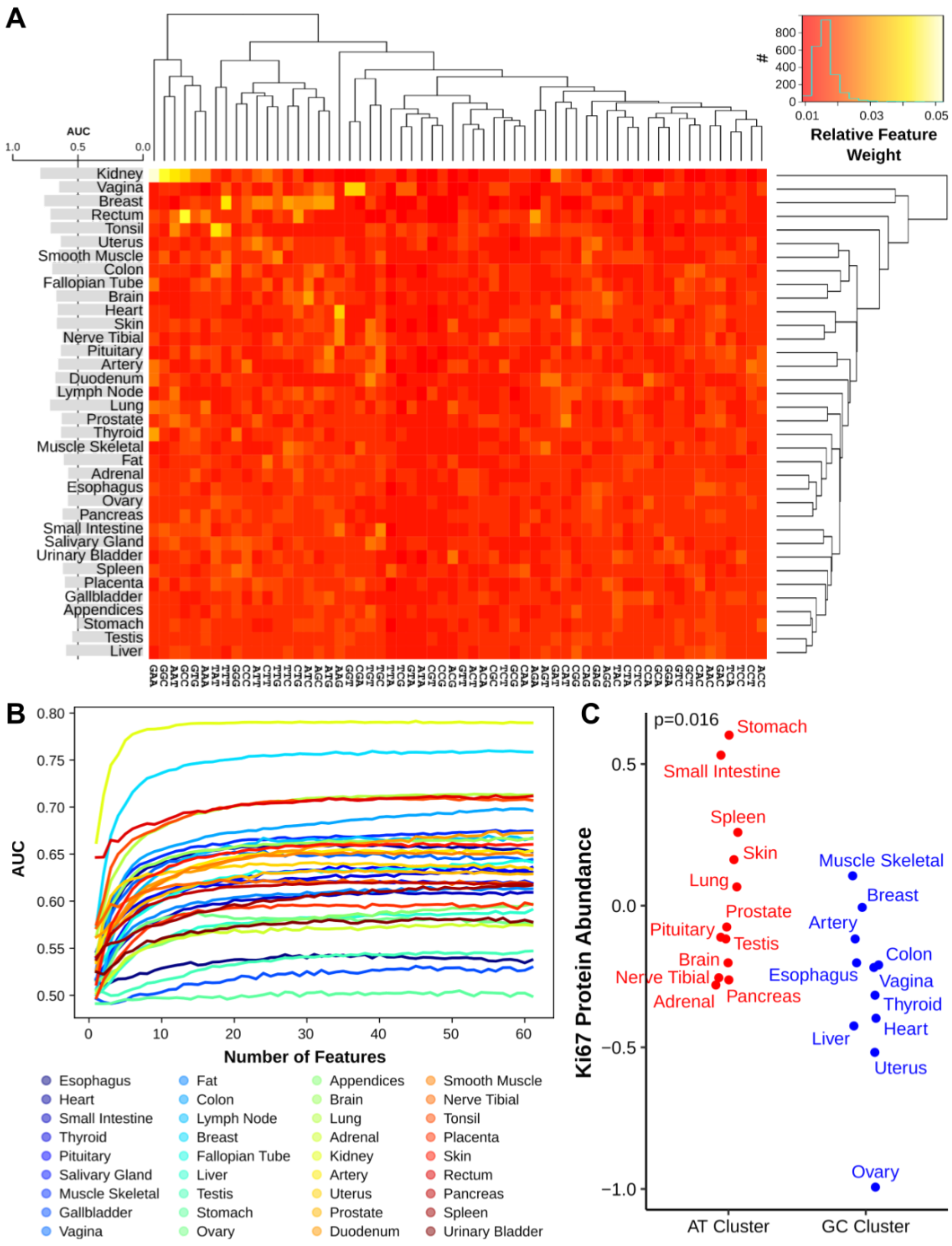

Figure S3. Random Forest models identify two clusters of human tissues with distinct codon signatures, related to Figure 2. (A) Based on the random forest classifiers, the heatmap shows the relative feature weights of every codon for each of the 36 human tissues, which measure the contribution of each codon in the decision trees. Codons and tissues were hierarchically clustered using euclidean distances and the complete-linkage method. The barplot on the left shows the mean AUC of the ROC curve of the RF model of each tissue. (B) Recursive Feature Elimination of the random forest model of each tissue. (C) Protein levels of the proliferation marker Ki67 across tissues in the GTEx dataset. Statistical difference between clusters (Fig. 2B) was determined by two-tailed T-test.

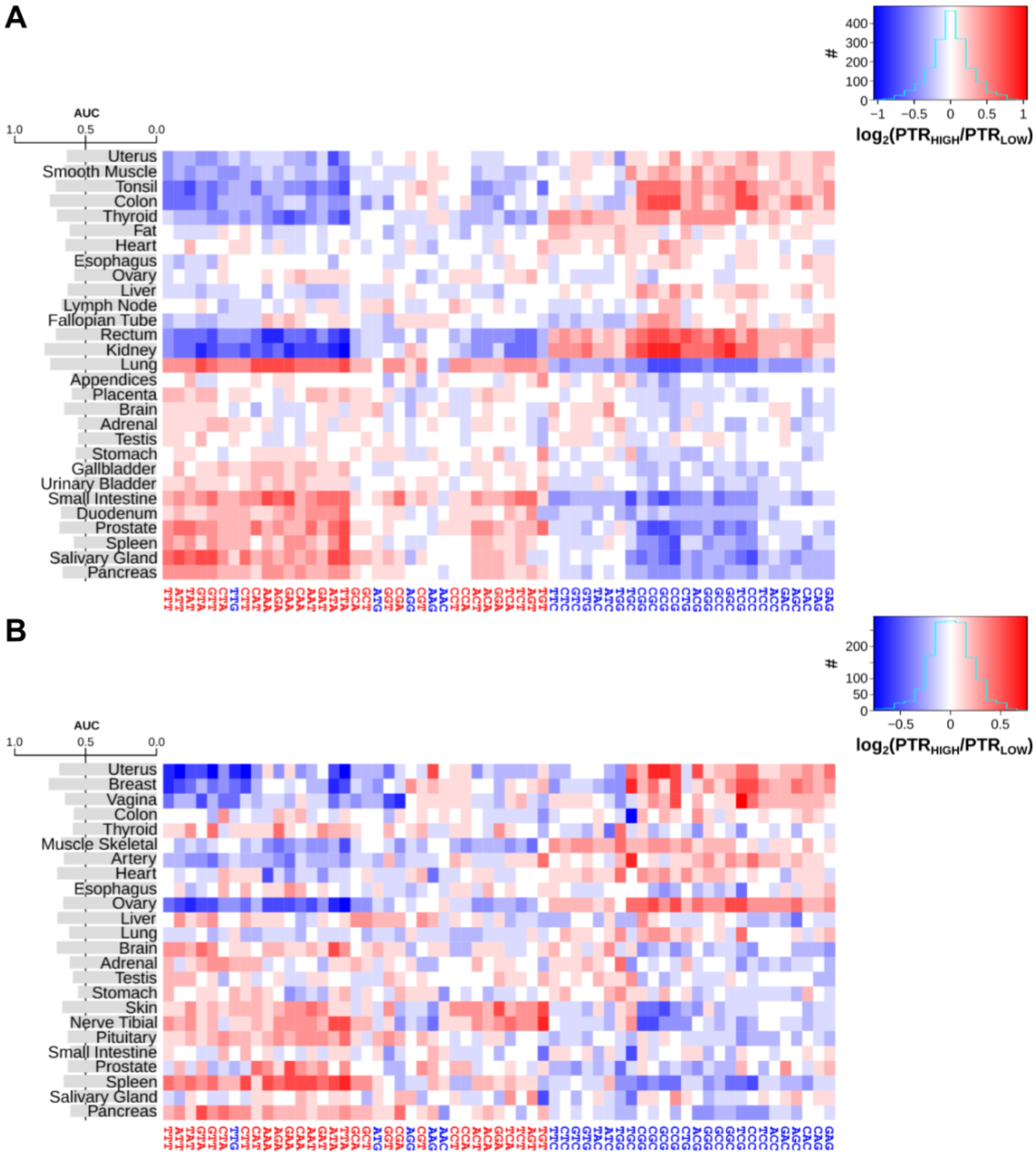

Figure S4. Random Forest models of HPA and GTEx datasets independently, related to Figure 2. (A) Ratios of the codon usage between high-PTR and low-PTR genes in each tissue of the HPA dataset. (B) Ratios of the codon usage between high-PTR and low-PTR genes in each tissue of the GTEx dataset. Codons and tissues follow the same order as in the clustering of Fig. 2B. The barplot on the left shows the mean AUC of the ROC curve of the RF model of each tissue.

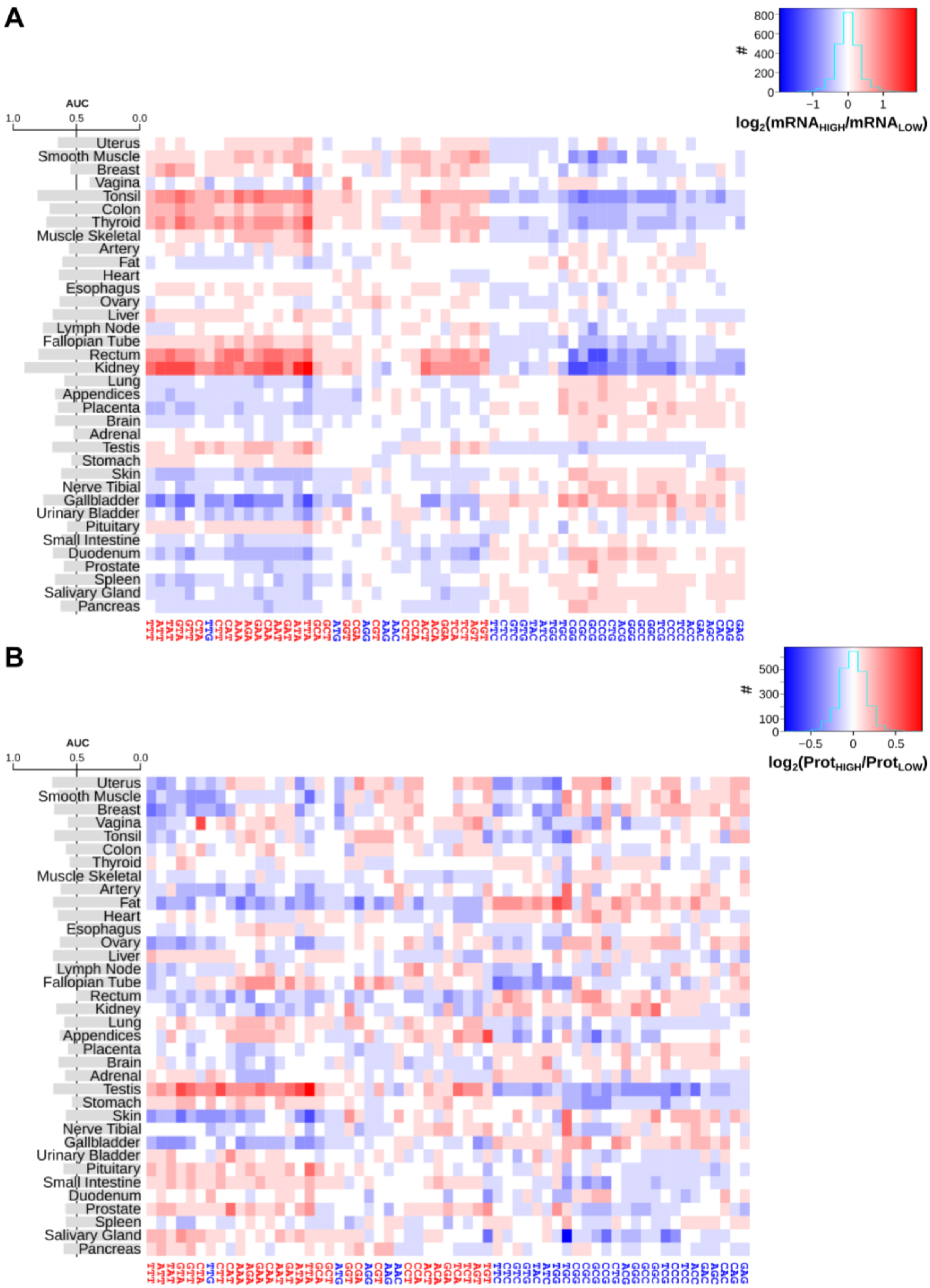

Figure S5. Random Forest models of tissue-specific mRNA and protein levels, related to Figure 2. (A) Ratios of the codon usage between high-mRNA and low-mRNA genes in each tissue. (B) Ratios of the codon usage between high-protein and low-protein genes in each tissue. Codons and tissues follow the same order as in the clustering of Fig. 2B. The barplot on the left shows the mean AUC of the ROC curve of the RF model of each tissue.

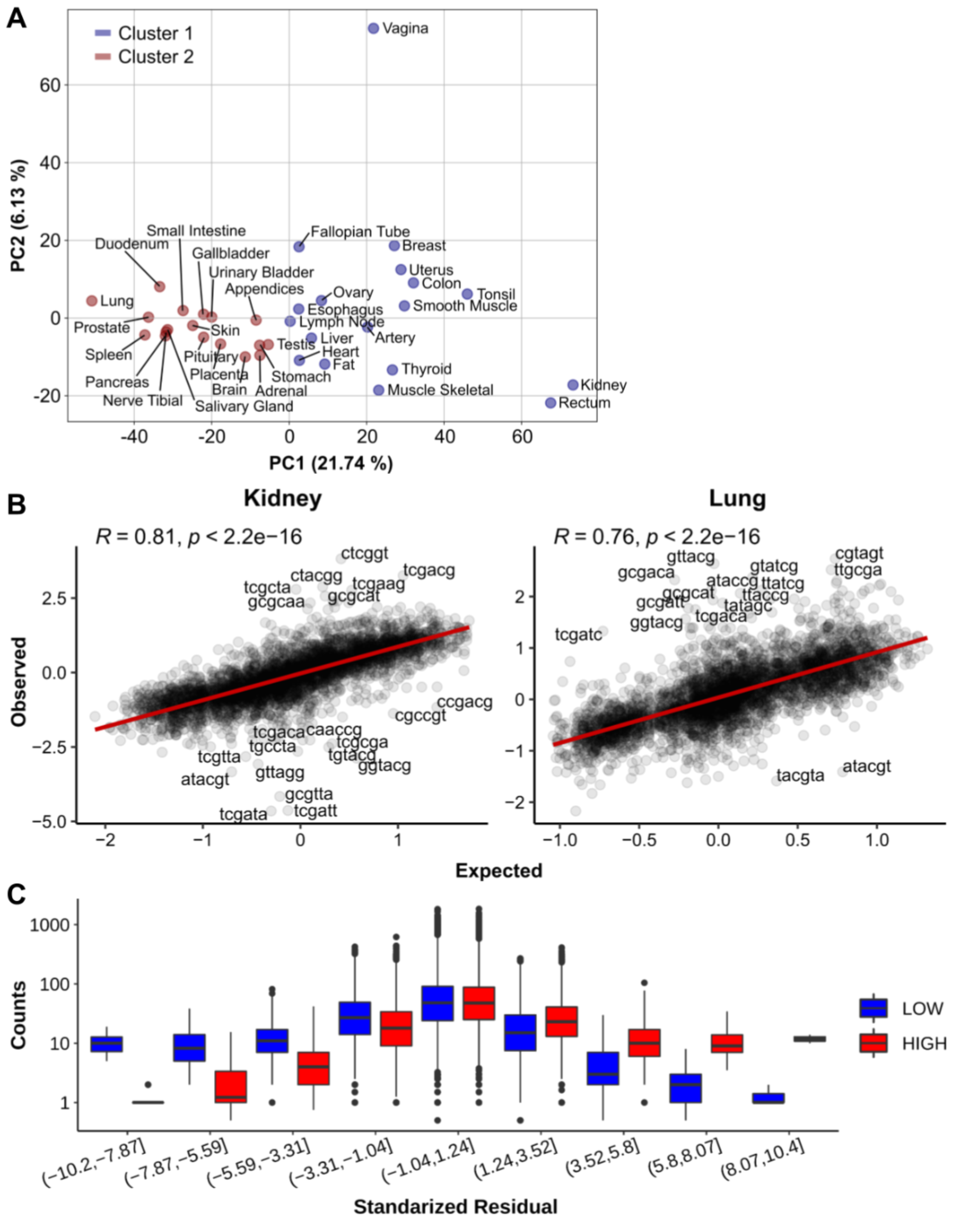

Figure S6. Differences between tissues are also observed at the codon pair level. (A) Principal Component Analysis (PCA) of the log-2-transformed ratios between high-PTR and low-PTR codon pair usage. The first axis completely separates the same two clusters detected in Fig. 2. (B) Linear regression in kidney and lung between the observed log-2-transformed codon pair ratios and the expected values based on the contribution of their constituent codons alone (see Methods). Codon pairs with a standardized residual higher than 4 are labeled. (C) For each standardized residual interval, number of codon pair counts in high-PTR and low-PTR gene sets. All tissues are merged together.

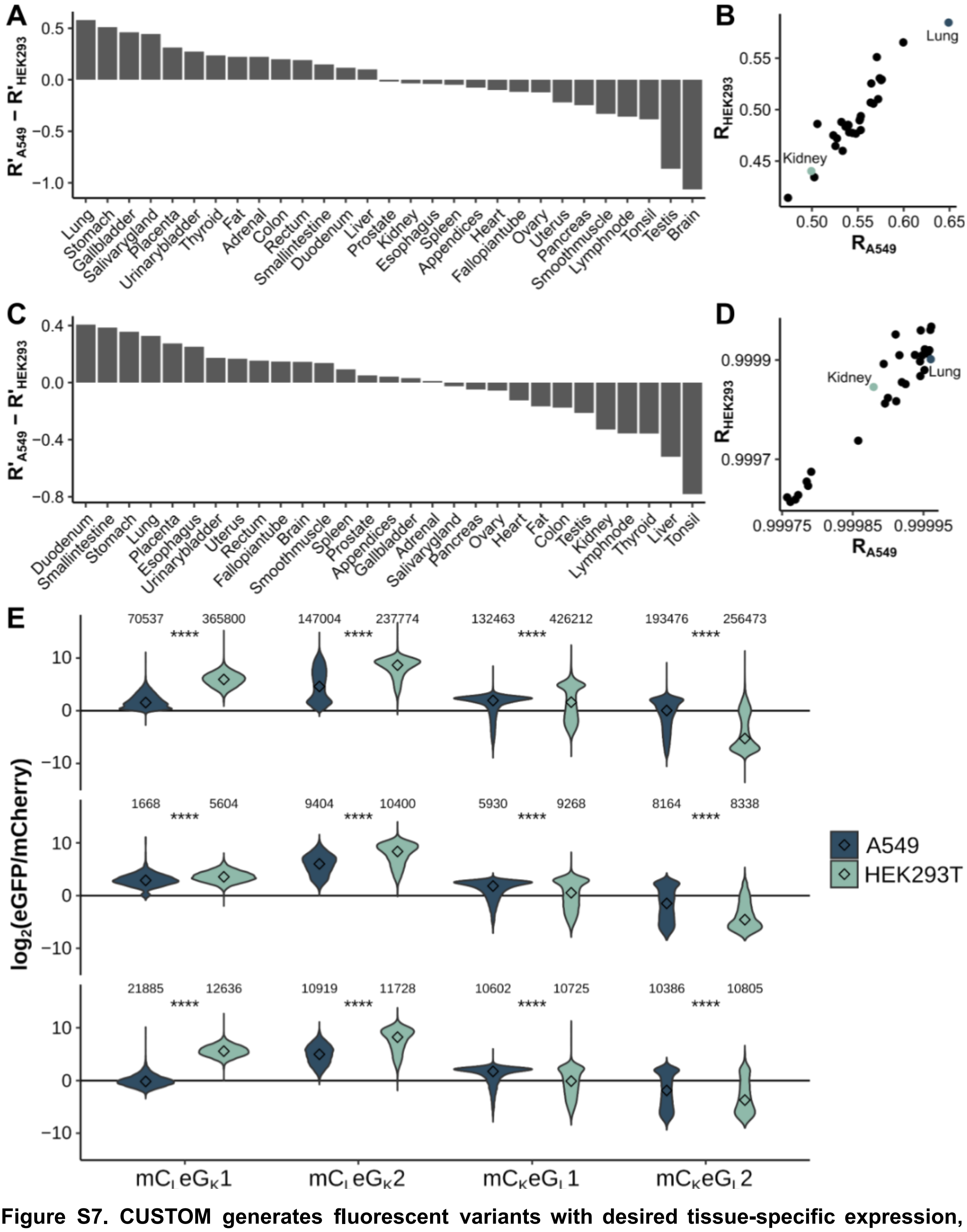

Figure S7. CUSTOM generates fluorescent variants with desired tissue-specific expression, related to Figure 3. (A) Difference between the standardized Spearman correlation of the proteomics profiles of A549 and HEK293 [2] against all tissues in the HPA [3]. (B) Spearman correlations of the proteomics profiles of A549 and HEK293 against all tissues in the HPA. (C) Difference between the standardized Pearson correlation of the proteomics-weighted codon usage profiles of A549 and HEK293 against all tissues in the HPA. (D) Pearson correlations of the proteomics-weighted codon usage profiles of A549 and HEK293 against all tissues in the HPA. (E) Ratios of eGFP and mCherry for each of the four constructs detected by flow cytometry. Panels correspond to different biological replicates. The number of cells within each group is specified. Center values represent the median. Statistical differences were determined by two-tailed Wilcoxon rank-sum test, and are denoted as follows: *$p \leq 0.05$, **$p \leq 0.01$, ***$p \leq 0.001$, ****$p \leq 0.0001$.

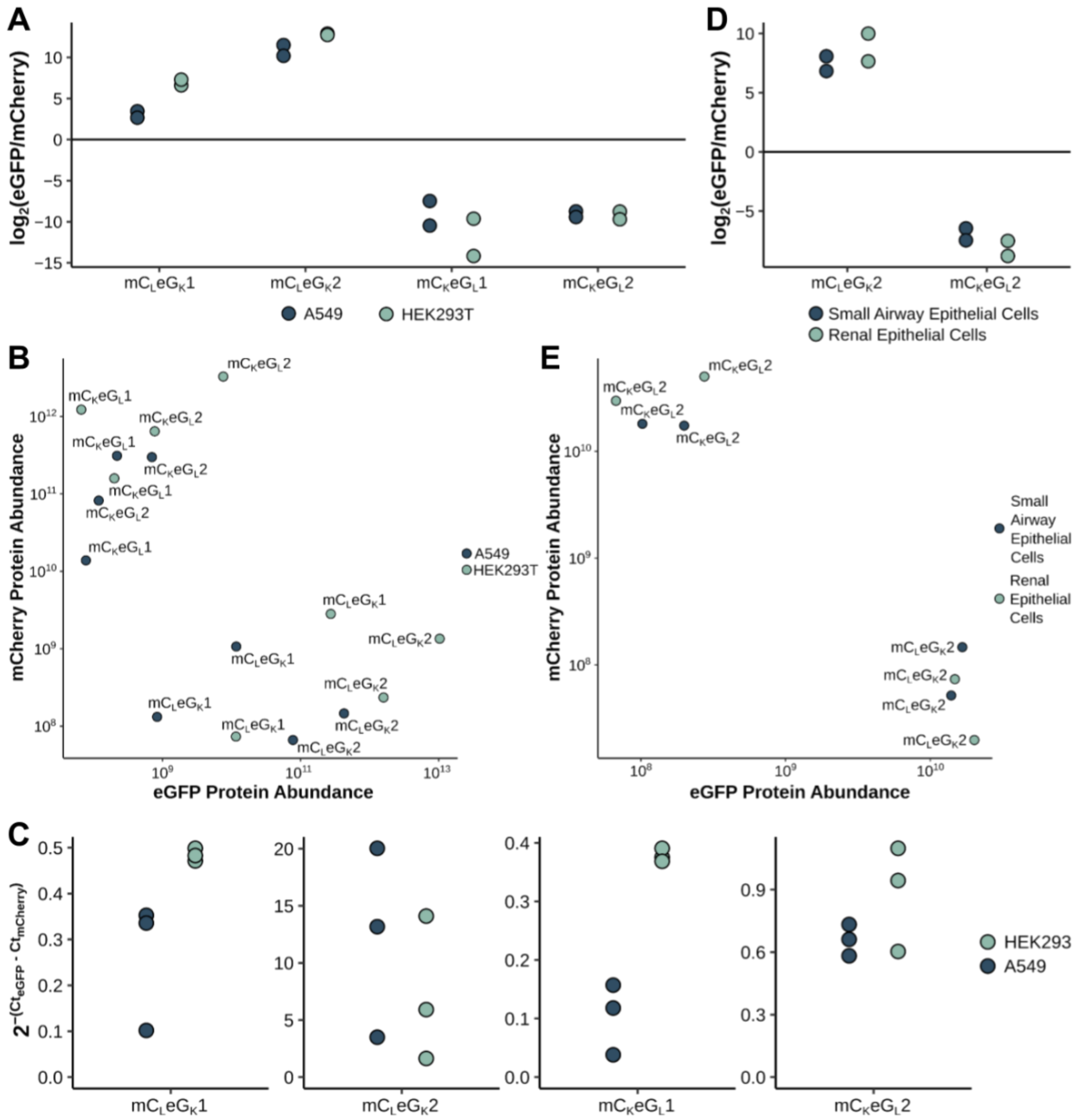

Figure S8. Proteomics and RT-qPCR of CUSTOM-optimized constructs. (A) Ratios of eGFP and mCherry for each of the four constructs in cell lines detected by targeted proteomics. Each dot corresponds to a biological replicate. (B) Absolute protein abundances of eGFP and mCherry for each of the four constructs in cell lines detected by targeted proteomics. (C) Ratios of eGFP and mCherry transcript abundance for each of the four constructs in cell lines detected by RT-qPCR. Each dot corresponds to a biological replicate. (D) Ratios of eGFP and mCherry for two of the constructs in primary cells detected by targeted proteomics. Each dot corresponds to a biological replicate. (E) Absolute protein abundances of eGFP and mCherry for two of the constructs in primary cells detected by targeted proteomics.

REFERENCES

1. Gremse M, Chang A, Schomburg I, Grote A, Scheer M, Ebeling C, et al. The BRENDA Tissue Ontology (BTO): the first all-integrating ontology of all organisms for enzyme sources. Nucleic Acids Res. 2011;39:D507-513.
2. Geiger T, Wehner A, Schaab C, Cox J, Mann M. Comparative proteomic analysis of eleven common cell lines reveals ubiquitous but varying expression of most proteins. Mol Cell Proteomics MCP. 2012;11:M111.014050.
3. Wang D, Eraslan B, Wieland T, Hallström B, Hopf T, Zolg DP, et al. A deep proteome and transcriptome abundance atlas of 29 healthy human tissues. Mol Syst Biol. John Wiley & Sons, Ltd; 2019;15:e8503.
