# Supplementary material for: Using protein-per-mRNA differences among human tissues in codon optimization
Source: Genome Biol. 2023 Feb 24;24:34. doi: 10.1186/s13059-023-02868-2 (PMC9951436; doi:10.1186/s13059-023-02868-2)
Supplement: Supplementary file 8 — Additional file 8. Peer review history. [file 13059_2023_2868_MOESM8_ESM.docx]

**Review history**

**First round of review**

**Reviewer 1**

In this manuscript, the authors have computationally derived tissue-specific codon optimality profiles using transcriptomic and proteomics datasets from the GTEx project and the Human Protein Atlas. Protein-to-mRNA (PTR) ratios were employed as proxy for translational efficiency in this analysis. The authors have employed the observed differences in codon preference or avoidance across tissues in the development of a codon optimization tool, CUSTOM to design genetic constructs for optimal expression of proteins in a tissue-specific manner. To validate the CUSTOM tool, the authors have generated variants of fluorescent proteins (eGFP and mCherry) for tissue-specific expression (kidney and lung) and studied their expression in corresponding cell line models (HEK293T and A549 respectively).
Comments to the authors:
The authors should address following concerns:
1) The authors have noted that tissue-specific codon usage derived from mRNA-sequence alone won't be able to capture the post-transcriptional regulation and accordingly used both transcriptomic and proteomic datasets to identify tissue-specific codon optimality profiles. In this regard, the authors could demonstrate and discuss the differences in the tissue-specific optimal codons identified by considering transcriptomic data alone vs the methodology employed by the authors. This analysis would substantiate the rationale for the methodology employed by the authors.
2) Experimental validation is not adequate to confirm the functionality of CUSTOM tool.
a. The authors have employed HEK293T and A549 cell lines as substitutes for kidney and lung tissues respectively. While the authors have compared proteomics data from the cell lines with respective tissues to demonstrate their suitability, it would have been ideal to perform these experiments in primary cells. For example, primary hepatocytes as surrogate for liver tissue. Considering that liver is an important target for several gene therapies, data generated from the primary hepatocytes will add more value to the data. Moreover, the authors didn't consider and noted how these cell lines are different in their codon usage from their respective primary tissues.
b. In the triplicate experiments performed in the cell line models, while the trends largely remained similar for the 4 constructs under study the actual fold changes appear to vary. It would be better to show combined data (from 3 experiments) as one figure and corresponding statistical evaluation in the manuscript. Data from individual experiments can be shown separately in the supplemental data.
c. The cell numbers varied significantly between experiments and the groups under comparison. The authors should comment on this observed variation and also relevant considerations taken in the statistical evaluation.
d. Lines 198 - 200: the data derived from experiments in cell lines is not sufficient to suggest that tissues like lung tolerate variability in codon usage better.
3) In the calculation of PTR ratios, the authors have taken protein secretion and degradation into consideration. In the same note, mRNA degradation rate/half file can also be expected to potentially impact PTR ratio calculations. It would be helpful to the readers to include relevant discussion in the manuscript.
4) In lines 88-90, the authors stated that high correspondence was observed between GTEX and Human Protein Atlas datasets. However, data in additional file 1 (Tab 6) indicates such high concordance is present for only 9 out of 17 common tissues in the datasets. The authors should comment on this and explain the difference between tissues that do not show high associations and those that show high correspondence. Also, it will be helpful to readers to clearly indicate the correspondence values in specific numbers rather than generic statement.
5) Supplemental figure 2B - It is difficult to distinguish between data points and asterisks added to indicate statistical significance.

**Reviewer 2**

In this manuscript, Hernandez-Alias and coauthors sought to determine whether protein-to-mRNA ratios measured across human tissues could be used for tissue-specific codon optimization. The answer was a clear "yes". This is a timely study that takes advantage of recent developments in quantitative proteomics and transcriptomics, and identifies distinct codon preferences across tissues. Functional genomic analyses are complemented by the development of software for tissue-specific codon optimization, and validated in an independent experiment using lung- and kidney-derived cell lines. As rightly suggested by the authors, these findings have potential applications in gene and mRNA therapy, in which the targeting of gene expression to a particular tissue is of vital importance.

Specific comments:

Gingold et al (Cell, 2014) also found a pattern of tissue-specific codon usage, based on a different type of data. Please cite this paper and discuss whether the tissue differences you identified are related to those described by Gingold et al.

The Random Forest models were trained to predict high-PTR and low-PTR genes in each tissue, i.e. the models predicted genes with high (or low) protein-to-mRNA ratios, but the validation experiment measured protein yield in each cell line (not normalized to mRNA). Wouldn't it be more appropriate to train and validate the model using the same type of data?

line 190: "the proteome of A549 clearly resembles that of lung, while HEK293 is a closer model to kidney (Supplemental Fig. 5A)" -> This figure is difficult to interpret. Please also show the correlation coefficient of A549 against all tissues in the HPA, and the correlation coefficient of HEK293 against all tissues in the HPA. It should also be noted that HEK293 were reported to be of neuronal origin.

line 199: "these observations suggest that A/T-ending codons are generally lower expressed than C/G-ending counterparts, but tissues like lung tolerate them better" -> can you show data to support this statement, for example a scatter plot from FACS? Currently only eGFP/mCherry ratios are shown, which do not allow to compare eGFP or mCherry expression across tissues.

line 554 (Figure 2): to show whether the conclusions are reproducible between GTEx and HPA datasets, it would be good to derive Figure 2B separately for the GTEx and HPA data, and show these two figures side by side in the supplement.

line 569 (Fig 3 legend): "Two additional replicates are shown in Supplemental Fig. 5A" -> should be "5B".

line 611 (Fig S5 legend): please explain that the top and bottom panels represent experimental replicates.

additional file 4: the sequences of only 4 variants are provided, and their names do not correspond to the names in Figure 3. Please provide the sequences of all variants used in the experimental validation, and use consistent names.

**Authors’ response to reviewers**

# Reviewer #1:

*In this manuscript, the authors have computationally derived tissue-speciﬁc codon optimality proﬁles using transcriptomic and proteomics datasets from the GTEx project and the Human Protein Atlas. Protein-to-mRNA (PTR) ratios were employed as proxy for translational eﬃciency in this analysis. The authors have employed the observed diﬀerences in codon preference or avoidance across tissues in the development of a codon optimization tool, CUSTOM to design genetic constructs for optimal expression of proteins in a tissue-speciﬁc manner. To validate the CUSTOM tool, the authors have generated variants of ﬂuorescent proteins (eGFP and mCherry) for tissue-speciﬁc expression (kidney and lung) and studied their expression in corresponding cell line models (HEK293T and A549 respectively).*

*Comments to the authors:*

*The authors should address following concerns:*

1) *The authors have noted that tissue-speciﬁc codon usage derived from mRNA-sequence alone won't be able to capture the post-transcriptional regulation and accordingly used both transcriptomic and proteomic datasets to identify tissue-speciﬁc codon optimality proﬁles. In this regard, the authors could demonstrate and discuss the diﬀerences in the tissue-speciﬁc optimal codons identiﬁed by considering transcriptomic data alone vs the methodology employed by the authors. This analysis would substantiate the rationale for the methodology employed by the authors.*

We thank the reviewer's comments on our work. We have followed their suggestion to analyze the codon optimality on mRNA-seq data alone, as well as on proteomics data alone (Fig. S5). We mention it in Results (2^nd^ section, 5^th^ paragraph): "Moreover, to disentangle the codon effects on mRNA and protein levels separately, we constructed a similar model using tissue-specific sets from either mRNA-seq or proteomic measurements alone (Additional file 2). As expected, since PTRs are defined as protein divided by transcript abundance, we observed an inverted codon signature related to mRNAs (Additional file 1: Fig. S5A, Additional file 3). Conversely, proteomics data alone led to a similar but less pronounced codon pattern as compared to PTRs (Additional file 1: Fig. S5B, Additional file 3), indicating that both mRNAs and proteins contribute to the observed PTR differences".

In addition, we have also validated that observed changes in protein levels across cell lines are not transcript-dependent (Results, 3^rd^ section, 3^rd^ paragraph): "Furthermore, to confirm that observed changes arise from differences in mRNA translation rather than at the transcript level, we quantified eGFP and mCherry transcript abundances by RT-qPCR (Additional file 5). In agreement, measured mRNA ratios inversely corresponded with protein ratios for three of the constructs (Additional file 1: Fig. S8C), which concurs with the previously observed mRNA model (Additional file 1: Fig. S5A)".

2) *Experimental validation is not adequate to conﬁrm the functionality of CUSTOM tool.*
   a. *The authors have employed HEK293T and A549 cell lines as substitutes for kidney and lung tissues respectively. While the authors have compared proteomics data from the cell lines with respective tissues to demonstrate their suitability, it would have been ideal to perform these experiments in primary cells. For example, primary hepatocytes as surrogate for liver tissue. Considering that liver is an important target for several gene therapies, data generated from the primary hepatocytes will add more value to the data. Moreover,*

*the authors didn't consider and noted how these cell lines are diﬀerent in their codon usage from their respective primary tissues.*

In the revised manuscript, we have both provided experimental evidence in primary cells and included the characterization of the codon usage of HEK293T and A549. On the first, the Results now indicate (3^rd^ section, 2^nd^ paragraph): "Based on available proteomics data of these cell lines [30], both the proteome and codon usage of A549 clearly resembled that of lung, while HEK293T was a closer model to kidney (Additional file 1: Fig. S7A-D)".

To replicate the validation in primary cells, we have expressed our four constructs in two independent batches of cells from lung (Human Small Airway Epithelial Cells, Lonza #CC-2547) and kidney (Human Renal Epithelial Cells, Lonza #CC-2556). While we appreciate the value that hepatocyte cells have in gene therapy, we focused on the aforementioned cells for three main reasons: (1) our four generated constructs are already optimized for lung and kidney tissues, (2) our results indicate that lung and kidney tissues have a higher tissue-specificity than liver based on their AUC (Fig. 2B), (3) the culture and transfection of hepatocyte cells is experimentally challenging and with extremely short viability.

The results with primary cells reproduce what we had previously observed in cell lines (Results, 3^rd^ section, 4^th^ paragraph): "As A549 is a cancer cell line and HEK293T is of adrenal origin [31], we sought to validate the construct expression in a more physiological model of tissue primary cells. The same tissue-specific pattern was consistently observed for all constructs except mC_K_eG_L_1 (where differences were less evident), both by flow cytometry (Figure 3E, Additional file 7) and targeted proteomics (Additional file 1: Fig. S8D-E, Additional file 5)"—mC_K_eG_L_1 is one of the two constructs with an eGFP optimized to lung and an mCherry optimized to kidney. We believe that this additional validation in primary cells now provides sufficient experimental evidence for usefulness of tissue-specific codon optimization.

b. *In the triplicate experiments performed in the cell line models, while the trends largely remained similar for the 4 constructs under study the actual fold changes appear to vary. It would be better to show combined data (from 3 experiments) as one ﬁgure and corresponding statistical evaluation in the manuscript. Data from individual experiments can be shown separately in the supplemental data.*

Following the reviewer's suggestion, we have now merged all the three replicates in the main figure, while also showing the data from individual replicates in Additional file 6 and Fig. S7E. To balance the contribution of each of the biological replicates with variable cell numbers (as discussed in the following comment), we have downsampled events to 1000 cells per group per replicate (Fig. 3C).

c. *The cell numbers varied signiﬁcantly between experiments and the groups under comparison. The authors should comment on this observed variation and also relevant considerations taken in the statistical evaluation.*

As the reviewer notes, the differences in transfection efficiency can be extremely variable, both in cell lines and primary cells, which we report on top of each violin plot of the corresponding figures (Fig. S7E, Fig. 3E). However, in terms of statistics, we perform Wilcoxon rank-sum tests, which is a non-parametric evaluation that does not take assumptions on the underlying data distribution and can deal with unequal sample sizes. Furthermore, to provide an orthogonal validation of our flow cytometry data, we have now measured protein abundances by targeted proteomics. We have also added an explicit comment in Results (3rd section, 3rd paragraph): "Moreover, while the efficiency of transfection was extremely variable between replicates, cell lines and constructs, we detected consistent significant differences in all three replicates (Additional file 1: Fig. S7E). Quantification of eGFP and mCherry by targeted proteomics showed the same tissue-specific pattern (Additional file 1: Fig. S8A-B, Additional file 5)".

d. *Lines 198 - 200: the data derived from experiments in cell lines is not suﬃcient to suggest that tissues like lung tolerate variability in codon usage better.*

To provide additional evidence that substantiates this claim, we have now included an additional panel in Fig. 3C to show the global variability of protein levels between the two cell lines. In this right panel, the reader can now observe that HEK293T has more bimodal eGFP/mCherry ratios than A549. In addition, we make available the raw fluorescence values of flow cytometry of cell lines and primary cells in Additional file 6 and 7, respectively.

Moreover, we provide additional validation by including an independent quantification of eGFP and mCherry by proteomics, which we report both as ratios and as absolute abundances (Fig. S8A-D).

3) *In the calculation of PTR ratios, the authors have taken protein secretion and degradation into consideration. In the same note, mRNA degradation rate/half ﬁle can also be expected to potentially impact PTR ratio calculations. It would be helpful to the readers to include relevant discussion in the manuscript.*

We followed the reviewer's suggestion and have now analyzed how mRNA half-lives impact PTR measurements. In the Results part (1^st^ section, 5^th^ paragraph) we added: "Similarly, no consistent association with mRNA degradation was detected using the mRNA half-life measurements of three different cell lines [24–26] either (Additional file 1: Fig. S2C, Additional file 2)". Furthermore, we have added a comment in the Discussion (4^th^ paragraph): "Moreover, while PTRs positively correlated with TE and results were not confounded by factors such as secretion or

mRNA and protein degradation, more direct readouts of TE will be possible as more ribosome profiling datasets of human tissues become available".

4) *In lines 88-90, the authors stated that high correspondence was observed between GTEX and Human Protein Atlas datasets. However, data in additional ﬁle 1 (Tab 6) indicates such high concordance is present for only 9 out of 17 common tissues in the datasets. The authors should comment on this and explain the diﬀerence between tissues that do not show high associations and those that show high correspondence. Also, it will be helpful to readers to clearly indicate the correspondence values in speciﬁc numbers rather than generic statement.*

This lack of concordance in eight tissues is mainly due to three factors: (1) low gene numbers, given that only genes detected in both mRNA-seq and proteomics and in both GTEx and HPA datasets can be compared; (2) PTR measurements accumulate noise from both mRNA-seq and proteomics (see lower albeit positive correlation in Fig. S1A), (3) the tissue types included in each dataset are not exactly the same, which can affect the definition of "tissue-specificity". To provide additional evidence of the concordance between datasets, we have therefore added the same analysis at both mRNA-seq and proteomic levels (Additional file 2). As the reviewer suggests, we specify these numbers in the text now (Results, 1^st^ section, 3^rd^ paragraph): "In particular, 16/17 tissues were significantly concordant at the mRNA level, 15/17 at the proteomic level, and 9/17 at the PTR level; only colon tissue was disagreeing at all levels".

Moreover, to analyze how the definition of "tissue-specificity" can affect this concordance analysis, we repeated the analysis with a less stringent definition of tissue-specific genes. The definition used in the paper is (see Methods): "(1) genes having a PTR fold change compared to the average of all other tissues larger than 2, (2) genes with the highest PTR among all tissues, (3) genes detected in at least 3 tissues in the dataset". In the analysis performed in this revision, we removed condition #2, which makes tissue-specific gene sets less dependent on the tissue types included in the dataset. With this less stringent definition, concordance between datasets is more apparent (Table R1). Some tissues (lung, salivary gland, stomach) remain discordant, which is likely due to the aforementioned error propagation effects, which makes PTRs more noisy than mRNA-seq or proteomics alone (Fig. S1A).

**Table R1. Concordance of protein sets between tissues in both HPA and GTEx.** High-PTR sets were defined as

(1) genes having a PTR fold change compared to the average of all other tissues larger than 2 and (2) genes detected in at least 3 tissues in the dataset, and vice versa for low-PTR sets. A binomial test is performed to assess whether the number of proteins in agreement (T) or not (F) between datasets is significantly concordant.

| **Tissue** | **PTR** | | | | **mRNA-seq** | | | | **Proteomics** | | | |
| --- | --- | --- | --- | --- | --- | --- | --- | --- | --- | --- | --- | --- |
|  | **T** | **F** | **Total** | **pval** | **T** | **F** | **Total** | **pval** | **T** | **F** | **Total** | **pval** |
| **Adrenal** | 209 | 88 | 3346 | 8.22E-13 | 414 | 59 | 2132 | 5.17E-67 | 611 | 96 | 4834 | 7.20E-93 |
| **Brain** | 673 | 81 | 4682 | 2.79E-117 | 868 | 33 | 3679 | 1.25E-211 | 1835 | 102 | 6746 | 0 |
| **Colon** | 121 | 93 | 3803 | 0.032 | 85 | 98 | 2183 | 0.849 | 182 | 137 | 4322 | 0.006 |
| **Uterus** | 125 | 48 | 3052 | 2.11E-09 | 209 | 23 | 1678 | 5.19E-39 | 544 | 72 | 4395 | 6.51E-91 |
| **Esophagus** | 137 | 75 | 3661 | 1.23E-05 | 294 | 22 | 2003 | 3.35E-62 | 352 | 61 | 4021 | 3.96E-51 |
| **Heart** | 420 | 94 | 4329 | 1.71E-50 | 872 | 18 | 3229 | 1.99E-231 | 1024 | 79 | 5619 | 1.47E-210 |
| **Liver** | 375 | 92 | 3988 | 7.04E-42 | 952 | 14 | 3508 | 1.05E-260 | 1260 | 90 | 5698 | 7.62E-265 |
| **Lung** | 163 | 163 | 4905 | 0.522 | 362 | 20 | 2061 | 1.17E-82 | 360 | 156 | 5334 | 6.69E-20 |
| **Ovary** | 226 | 117 | 4198 | 2.05E-09 | 452 | 8 | 2402 | 1.60E-122 | 369 | 202 | 4825 | 1.31E-12 |
| **Pancreas** | 271 | 111 | 4407 | 7.36E-17 | 496 | 16 | 2623 | 6.48E-125 | 867 | 146 | 5851 | 1.30E-125 |
| **Prostate** | 132 | 56 | 3507 | 1.47E-08 | 188 | 26 | 1612 | 8.73E-32 | 322 | 77 | 4006 | 5.12E-37 |
| **Salivarygland** | 141 | 156 | 3948 | 0.823 | 229 | 61 | 2550 | 2.40E-24 | 276 | 131 | 4715 | 2.70E-13 |
| **Smallintestine** | 174 | 81 | 3462 | 2.86E-09 | 224 | 56 | 2347 | 2.88E-25 | 713 | 113 | 4878 | 1.60E-107 |
| **Spleen** | 207 | 34 | 3103 | 9.68E-32 | 618 | 3 | 2264 | 4.59E-180 | 887 | 82 | 5067 | 1.03E-171 |
| **Stomach** | 118 | 127 | 3184 | 0.738 | 256 | 37 | 1827 | 9.33E-42 | 412 | 100 | 4231 | 2.81E-46 |
| **Testis** | 518 | 89 | 4555 | 7.83E-75 | 1004 | 10 | 3948 | 1.74E-282 | 981 | 101 | 5831 | 5.28E-182 |
| **Thyroid** | 175 | 55 | 3726 | 4.56E-16 | 203 | 32 | 2372 | 6.68E-32 | 357 | 66 | 4752 | 1.07E-49 |

However, re-building the model with this other tissue-specificity definition does not change nor increase at all the codon optimality signal (Fig. R1). Therefore, while we make this analysis available in the revision, we kept the original definition in the manuscript for coherence with the rest of analyses and experiments performed.

In addition, we now also stress the concordance between datasets by deriving the codon usage model from HPA and GTEx datasets independently (Results, 2^nd^ section, 5^th^ paragraph): "To further assess the concordance between GTEx and HPA datasets, we also reproduced the same analyses with the high-PTR vs low-PTR sets of each dataset separately (Additional file 2). With few differences, both datasets showed a similar A/T-ending vs G/C-ending signature across tissues (Additional file 1: Fig. S4, Additional file 3)".

**Figure R1. Random Forest models of tissue-specific PTRs.** Ratios of the codon usage between high-PTR and low-PTR genes in each tissue. High-PTR sets were defined as (1) genes having a PTR fold change compared to the average of all other tissues larger than 2 and (2) genes detected in at least 3 tissues in the dataset, and vice versa for low-PTR sets. Codons and tissues follow the same order as in the clustering of Fig. 2B. The barplot on the left shows the mean AUC of the ROC curve of the RF model of each tissue.

5) *Supplemental ﬁgure 2B - It is diﬃcult to distinguish between data points and asterisks added to indicate statistical signiﬁcance.*

We have changed the color of asterisks to gray.

# Reviewer #2:

*In this manuscript, Hernandez-Alias and coauthors sought to determine whether protein-to-mRNA ratios measured across human tissues could be used for tissue-speciﬁc codon optimization. The answer was a clear "yes". This is a timely study that takes advantage of recent developments in quantitative proteomics and transcriptomics, and identiﬁes distinct codon preferences across tissues. Functional genomic analyses are complemented by the development of software for tissue-speciﬁc codon optimization, and validated in an independent experiment using lung- and kidney-derived cell lines. As rightly suggested by the authors, these ﬁndings have potential applications in gene and*

*mRNA therapy, in which the targeting of gene expression to a particular tissue is of vital importance.*

We thank the reviewer's encouragement on the relevance and utility of our manuscript.

*Speciﬁc comments:*

*Gingold et al (Cell, 2014) also found a pattern of tissue-speciﬁc codon usage, based on a diﬀerent type of data. Please cite this paper and discuss whether the tissue diﬀerences you identiﬁed are related to those described by Gingold et al.*

In Gingold et al (Cell, 2014), the authors observed that most differences in codon usage across gene sets and in tRNA expression across patients and cell lines are related to the regulation of proliferation and differentiation. We now cite this article and analyze how the detected tissue-specificity is associated with the proliferative state of tissues (Results, 2^nd^ section, 4^th^ paragraph): "In agreement with studies showing that the A/T-vs-G/C pattern is associated with proliferation [10,27], we also detected that the A/T-ending cluster was significantly more proliferative than G/C-ending one, based on the proliferation marker Ki67 [28] (Additional file 1: Fig. S3C)".

*The Random Forest models were trained to predict high-PTR and low-PTR genes in each tissue, i.e. the models predicted genes with high (or low) protein-to-mRNA ratios, but the validation experiment measured protein yield in each cell line (not normalized to mRNA). Wouldn't it be more appropriate to train and validate the model using the same type of data?*

While codon usage can affect gene expression at multiple levels, we focused in this manuscript on the level of translation, and therefore we used PTRs to estimate translation efficiency. However, we agree that this led to a discrepancy between training and validation. Therefore, we now also provide the results of trained models using either mRNA-seq and proteomics (Results, 2^nd^ section, 5^th^ paragraph): "Moreover, to disentangle the codon effects on mRNA and protein levels separately, we constructed a similar model using tissue-specific sets from either mRNA-seq or proteomic measurements alone (Additional file 2). As expected, since PTRs are defined as protein divided by transcript abundance, we observed an inverted codon signature related to mRNAs (Additional file 1: Fig. S5A, Additional file 3). Conversely, proteomics data alone led to a similar but less pronounced codon pattern as compared to PTRs (Additional file 1: Fig. S5B, Additional file 3), indicating that both mRNAs and proteins contribute to the observed PTR differences".

Furthermore, to better compare the validation results to the PTR-trained model, we have performed RT-qPCR of transfected cell lines (Results, 3^rd^ section, 3^rd^ paragraph): "Furthermore, to confirm that observed changes arise from differences in mRNA translation rather than at the

transcript level, we quantified eGFP and mCherry transcript abundances by RT-qPCR (Additional file 5). In agreement, measured mRNA ratios inversely corresponded with protein ratios for three of the constructs (Additional file 1: Fig. S8C), which concurs with the previously observed mRNA model (Additional file 1: Fig. S5A)".

*line 190: "the proteome of A549 clearly resembles that of lung, while HEK293 is a closer model to kidney (Supplemental Fig. 5A)" -> This ﬁgure is diﬃcult to interpret. Please also show the correlation coeﬃcient of A549 against all tissues in the HPA, and the correlation coeﬃcient of HEK293 against all tissues in the HPA. It should also be noted that HEK293 were reported to be of neuronal origin.*

We now report the correlation coefficients of A549 and HEK293T against all tissues in Fig. S7B. As suggested by reviewer 1, we also provide the correlations at the level of codon usage between cells and tissues (Fig. S7C-D).

Moreover, to validate the observations in a more realistic system, we have performed flow cytometry and proteomics of two independent batches of primary cells from lung (Human Small Airway Epithelial Cells, Lonza #CC-2547) and kidney (Human Renal Epithelial Cells, Lonza #CC-2556) (Results, 3^rd^ section, 4^th^ paragraph): "As A549 is a cancer cell line and HEK293T is of adrenal origin [31], we sought to validate the construct expression in a more physiological model of tissue primary cells. The same tissue-specific pattern was consistently observed for all constructs except mC_K_eG_L_1 (where differences were less evident), both by flow cytometry (Figure 3E, Additional file 7) and targeted proteomics (Additional file 1: Fig. S8D-E, Additional file 5)"—mC_K_eG_L_1 is one of the two constructs with an eGFP optimized to lung and an mCherry optimized to kidney.

As mentioned by the reviewer, we also report that HEK293 are of adrenal origin (see above). Actually, as discussed in the article we cite, adrenal cells are close to neuronal cells and can easily enter into neuronal differentiation.

*line 199: "these observations suggest that A/T-ending codons are generally lower expressed than C/G-ending counterparts, but tissues like lung tolerate them better" -> can you show data to support this statement, for example a scatter plot from FACS? Currently only eGFP/mCherry ratios are shown, which do not allow to compare eGFP or mCherry expression across tissues.*

To provide additional evidence that substantiates this claim, we have now included an additional panel in Fig. 3C to show the global variability of protein levels between the two cell lines. In this right panel, the reader can now observe that HEK293T has more bimodal eGFP/mCherry ratios than A549. In addition, we make available the raw fluorescence values of flow cytometry of cell lines and primary cells in Additional file 6 and 7, respectively.

Moreover, we provide additional validation by including an independent quantification of eGFP and mCherry by proteomics, which we report both as ratios and as absolute abundances (Fig. S8A-D).

*line 554 (Figure 2): to show whether the conclusions are reproducible between GTEx and HPA datasets, it would be good to derive Figure 2B separately for the GTEx and HPA data, and show these two ﬁgures side by side in the supplement.*

As suggested by the reviewer, we have built the model for each of the two datasets separately (Results, 2^nd^ section, 5^th^ paragraph): "To further assess the concordance between GTEx and HPA datasets, we also reproduced the same analyses with the high-PTR vs low-PTR sets of each dataset separately (Additional file 2). With few differences, both datasets showed a similar A/T-ending vs G/C-ending signature across tissues (Additional file 1: Fig. S4, Additional file 3)".

*line 569 (Fig 3 legend): "Two additional replicates are shown in Supplemental Fig. 5A"*

*-> should be "5B".*

We thank the reviewer for spotting the error, which is now corrected.

*line 611 (Fig S5 legend): please explain that the top and bottom panels represent experimental replicates.*

We now indicate in the legend (Fig. S7E): "Panels correspond to different experimental replicates".

*additional ﬁle 4: the sequences of only 4 variants are provided, and their names do not correspond to the names in Figure 3. Please provide the sequences of all variants used in the experimental validation, and use consistent names.*

We have now corrected the error in this supplementary table and included the sequence names as they appear in Fig. 3.

**Second round of review**

**Reviewer 1**

Thank you for addressing the comments.

**Reviewer 2**

I have no further comments.
